# Subcutaneous versus intravenous trastuzumab for HER2-positive breast cancer: a global systematic review and meta-analysis with a cost-minimization analysis from the Chinese healthcare system perspective

**DOI:** 10.3389/fphar.2025.1730175

**Published:** 2026-01-07

**Authors:** Zheng Zeng, Li Zhong, Ling Zhu, Luqin Liao, Zefeng Zhu, Yuening Cao, Da Zheng, Guidong Huang, Wei Chen, Lin Zhang

**Affiliations:** 1 The Department of Pharmacy, The First Affiliated Hospital of Guilin Medical University, Guilin, China; 2 College of Pharmacy, Guilin Medical University, Guilin, China; 3 Phase I Clinical Research Center of Drugs, The First Affiliated Hospital of Guilin Medical University, Guilin, China; 4 Department of Breast and Thyroid Surgery, The First Affiliated Hospital of Guilin Medical University, Guilin, China

**Keywords:** HER2-positive breast cancer, intravenous administration, patient preference, subcutaneous injection, systematic review, trastuzumab

## Abstract

**Background:**

Subcutaneous (SC) trastuzumab offers a more convenient alternative to intravenous (IV) administration for HER2-positive breast cancer, potentially improving healthcare efficiency and patient experience. Although SC trastuzumab was approved in Europe in 2013 and in the United States in 2019, it only became available in China in 2022, highlighting the need to synthesize global evidence for regions where SC adoption is recent.

**Methods:**

A systematic search of PubMed, Embase, Web of Science, and the Cochrane Library through 20 March 2025, identified studies comparing SC and IV trastuzumab. Meta-analyses were performed using random- or fixed-effects models to evaluate pathological complete response (pCR), event-free survival (EFS), adverse events, serious adverse events, and patient preference. A cost-minimization analysis (CMA) was additionally performed from the perspective of the Chinese healthcare system.

**Results:**

Nine studies were included. SC trastuzumab demonstrated comparable pCR (OR = 1.11, 95% CI: 0.86–1.42) and EFS (HR = 0.96, 95% CI: 0.78–1.19) to IV administration. SC was associated with a higher incidence of mild-to-moderate local reactions (OR = 1.59, 95% CI: 1.38–1.84) but no significant difference in serious adverse events (OR = 1.37, 95% CI: 0.94–1.99). Patient preference strongly favored SC (OR = 63.02, 95% CI: 34.43–115.34). Cost-minimization analysis showed that 18 cycles of SC trastuzumab (100,597 CNY) reduced costs by approximately 7% compared with the IV originator (108,032 CNY) and were generally comparable to domestic biosimilars, which ranged from 79,432 to 101,817 CNY.

**Conclusion:**

SC trastuzumab demonstrates comparable clinical outcomes to IV administration, with a marked patient preference advantage and potential cost savings compared with the IV originator and domestic biosimilars. These findings are particularly relevant to healthcare systems where SC formulations are newly introduced, providing timely evidence to guide patient-centered clinical decision-making.

**Systematic Review Registration:**

https://www.crd.york.ac.uk/prospero/display_record.php?RecordID=637674, identifier CRD42025637674.

## 1 Introduction

Breast cancer remains a leading cause of cancer-related mortality worldwide (Bray et al., 2024). In China, approximately 15%–20% of cases are human epidermal growth factor receptor 2 (HER2)-positive, an aggressive subtype requiring targeted therapy (Lin et al., 2017). Trastuzumab serves as the cornerstone of treatment, significantly improving survival outcomes in both early and advanced stages (Gradishar et al., 2024; Ba et al., 2012).

Traditionally, trastuzumab is administered via intravenous (IV) infusion. While effective, IV administration imposes significant time burdens on patients and resource demands on healthcare systems. To address these limitations, a subcutaneous (SC) formulation was developed, offering a fixed dose that allows for rapid administration without the need for venous access (Gao et al., 2021). While widely adopted globally, SC trastuzumab was only approved in China in 2022, meaning clinical integration is still in the early stages.

Beyond clinical efficacy and safety, economic considerations are pivotal. Although international studies suggest SC administration reduces healthcare costs (Lopez-Vivanco et al., 2017; O'Brien et al., 2019; Rojas et al., 2020; Lee and Cheng, 2023), the unique characteristics of the Chinese healthcare system—including specific insurance policies and price variability between imported and domestic IV products—warrant a dedicated evaluation. Therefore, this study aimed to perform a systematic review and meta-analysis to comprehensively compare subcutaneous and intravenous trastuzumab in HER2-positive breast cancer regarding efficacy, safety, and patient preference. Furthermore, based on the synthesized clinical evidence, we conducted a comparative cost analysis from the perspective of the Chinese healthcare system to provide comprehensive evidence for clinical and policy decision-making.

## 2 Materials and methods

### 2.1 Inclusion and exclusion criteria

#### 2.1.1 Inclusion criteria

(1) Research subjects: Patients diagnosed with HER2-positive breast cancer in accordance with the “Breast Cancer Diagnosis and Treatment Guidelines (2022 Edition); (2) Intervention measures: Trastuzumab administration (via subcutaneous injection), either as monotherapy or in combination with other therapies, without restriction on the duration of treatment or dosage; (3) Control Measures: Trastuzumab for injection (intravenous administration), administered as monotherapy or in combination with other agents, without restriction on the duration of treatment or dosage; (4) Outcome indicators: The efficacy outcomes encompass the pathological complete response rate (pCR), disease-free survival (DFS), progression-free survival (PFS), overall survival (OS), and objective response rate (ORR). Safety outcomes include the incidence of serious adverse events (SAEs) and the overall incidence of adverse events (AEs); (5) Language: Chinese or English; (6) Literature types: Randomized controlled trials, cohort studies, and case-control studies.

#### 2.1.2 Exclusion criteria

(1) Articles that are repeatedly published in multiple journals or platforms; (2) Studies lacking outcome indicators of effectiveness or safety; (3) Studies in which critical outcome measures cannot be reliably extracted; (4) Non-primary research literature (including reviews, case reports, animal studies, conference abstracts, letters, dissertations, and other materials that have not undergone peer review); (5) The full text is unavailable; (6) The quality of the literature is suboptimal; (7) Non-Chinese and English literature.

### 2.2 Literature search strategies

This systematic review and meta-analysis was conducted in strict accordance with the guidelines of the Preferred Reporting Items for Systematic Reviews and Meta-Analyses (PRISMA). The research protocol has been registered with the international prospective registration platform PROSPERO, registration number CRD42025637674. A combination of subject headings and free-text terms was employed to conduct a computerized search in databases including PubMed, Web of Science, Cochrane Library, and Embase. The search time frame spanned from the inception of each database to 20 March 2025. Key search terms included: “HER2-positive breast cancer,” “intravenous administration,” “subcutaneous administration,” and “trastuzumab.” The search strategies employed for all databases are comprehensively detailed in Supplementary Table S1.

### 2.3 Literature screening and data extraction

Literature screening and quality evaluation were independently conducted by two researchers. EndNote X9 software was utilized for literature management, with duplicate records excluded via both automated and manual de-duplication processes. Initially, titles and abstracts were reviewed to exclude studies that did not meet the inclusion criteria. Subsequently, full texts were meticulously examined to identify eligible studies. In cases of disagreement, consensus was achieved through discussion or consultation with a third researcher. Data were organized using Excel 2019, and information from each study was extracted using a standardized form, which included details such as the article title, first author, publication date, study design, subject inclusion criteria, sample size, intervention and control measures, outcome indicators, etc. For incomplete data, authors were contacted to obtain missing information. The final results were cross-verified by two researchers, and any discrepancies were resolved through negotiation.

### 2.4 Assessment of literature quality

The Cochrane risk of bias assessment tool (Higgins et al., 2011) was utilized to evaluate the risk of bias in the included randomized controlled trials. This evaluation encompassed seven specific domains: random sequence generation, allocation concealment, blinding of participants and personnel, blinding of outcome assessors, incomplete outcome data, selective reporting, and other potential sources of bias. Subsequently, conduct three types of assessments: “low risk”, “high risk”, and “indeterminate”. In case-control and cohort studies, the Newcastle-Ottawa Scale (NOS) (Luchini et al., 2017) was utilized to evaluate study quality across three domains: selection of participants, comparability between groups, and outcome assessment. Bias assessment followed a semi-quantitative star system, wherein each criterion could receive up to one star, except for group comparability, which could receive up to two stars. The maximum score was nine stars, with higher scores indicating greater study quality. Studies scoring 0–3 stars were classified as low-quality, those scoring 4–6 stars as moderate-quality, and those scoring 7–9 stars as high-quality.

### 2.5 Statistical analysis

Data processing was conducted using Review Manager 5.4 software. Time-to-event data were analyzed using hazard ratios (HR) with corresponding 95% confidence intervals (95% CI). Count data were reported as odds ratios (OR) with corresponding 95% CI, while continuous data were presented as mean differences (MD) along with 95% CI. For survival analysis outcomes, the natural logarithm of HR [ln (HR)] and its standard error were calculated based on the reported HR and 95% CI from individual studies, then pooled using the generic inverse variance approach. The extent of heterogeneity among studies was assessed using the P value and I^2^ statistic. A P value less than 0.1 indicated significant heterogeneity. An I^2^ value of 50% served as the threshold for substantial heterogeneity. Specifically, an I^2^ ≥ 50% suggested the presence of notable heterogeneity, whereas 0%–40% indicated negligible heterogeneity, 30%–60% indicated moderate heterogeneity, and 50%–90% indicated substantial heterogeneity. Therefore, the degree of heterogeneity could be effectively evaluated and quantified using the P value and I^2^ statistic (Migliavaca et al., 2022). When I^2^ < 50% or P ≥ 0.1, it indicated that heterogeneity was not significant, and a fixed-effect model was applied; when P < 0.1 or I^2^ ≥ 50%, it indicated significant heterogeneity, and a random-effects model was used. Sensitivity analysis or subgroup analysis was performed using the sequential exclusion method to identify potential sources of heterogeneity. If the number of included studies in a group exceeded 10, an inverted funnel plot was utilized to assess potential publication bias.

### 2.6 GRADE evidence grading system

Evidence quality was evaluated using GRADE profiler 3.6, and the evidence was classified into four grades: high, moderate, low, and very low. The evaluation contents included: risk of bias, inconsistency, indirectness, imprecision, and publication bias.

### 2.7 Pharmacoeconomic evaluation

This study aimed to evaluate the economic differences between SC and IV trastuzumab in China. Model Selection Protocol Based on Guidelines The choice of the economic evaluation framework was strictly guided by national and international pharmacoeconomic standards, specifically the China Guidelines for Pharmacoeconomic Evaluations (2020 Edition) (Liu and Wu, 2020), International Society for Pharmacoeconomics and Outcomes Research (ISPOR), and National Institute for Health and Care Excellence (NICE) recommendations. A conditional decision rule was applied as follows: If the systematic review and meta-analysis indicate no significant differences in efficacy and safety between SC and IV administration, a minimal cost analysis will be conducted; if significant differences exist, a cost-utility analysis will be performed to integrate both cost and clinical outcomes.

#### 2.7.1 Perspective and cost estimation

The analysis was conducted from the perspective of the Chinese healthcare system, including only direct medical costs; indirect and intangible costs were excluded. The analysis horizon corresponded to a standard 1-year trastuzumab treatment (18 cycles, every 3 weeks). All costs were expressed in 2025 Chinese Yuan (CNY), and no discounting was applied due to the 1-year time horizon.

Costs comprised:

1. Drug acquisition costs: IV trastuzumab formulations included the imported originator product (Herceptin®) and multiple domestically manufactured biosimilars, which differ substantially in price in China. SC trastuzumab was administered at a fixed dose of 600 mg, whereas IV dosing followed a weight-based regimen (initial 8 mg/kg, maintenance 6 mg/kg). The originator IV product contains preservatives, allowing multi-use after reconstitution, whereas biosimilars lack preservatives, requiring disposal of remaining solution. Drug costs were calculated based on actual vial usage and patient body weight, with average height (1.58 m) and weight (59 kg) of Chinese women derived from the China Nutrition and Chronic Disease Report (2020) (Pan et al., 2021).
2. Administration and service costs: included preparation, reconstitution, dilution, infusion or injection consumables, personnel time, infusion-related charges, and nursing fees.
3. Monitoring and laboratory costs: included routine laboratory tests and cardiac function monitoring during treatment.

#### 2.7.2 Data sources

Drug acquisition costs were obtained from the National Reimbursement Drug List (NRDL) of China and procurement records from a tertiary hospital. Administration, drug management, and monitoring costs were extracted from the hospital electronic medical record system and fee schedule. All costs were accumulated over the full treatment cycle according to actual usage.

#### 2.7.3 Sensitivity analysis

To evaluate the robustness of results, one-way sensitivity analyses were performed to examine the impact of key parameters on cost differences, including drug price, patient body weight, consumable costs, and drug management fees. Each parameter was varied by ±20% of its base-case value.

## 3 Results

### 3.1 The outcome of the literature screening process

**FIGURE 1:**
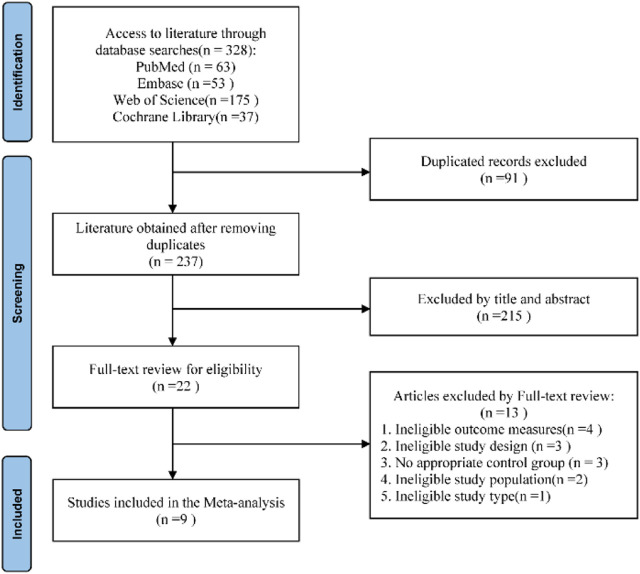
Literature screening process and results.

By the retrieval strategy, an initial set of 328 literatures was identified. After removing duplicate entries using EndNote X9 literature management software, a final count of 237 unique literatures was obtained. Following an in-depth review and evaluation of the abstracts, titles, and full texts, a total of nine literatures were ultimately selected for inclusion. The detailed process and outcomes of the literature screening are illustrated in Figure 1.

### 3.2 The fundamental attributes of the literature

A total of nine studies (Ismael et al., 2012; Pivot et al., 2013; Pivot et al., 2014; Pivot et al., 2017a; Pivot et al., 2017b; Jackisch et al., 2019; O'Shaughnessy et al., 2021; Tan et al., 2021; Pellegrino et al., 2023) were included in this review, all of which were publicly published. These studies were published between 2012 and 2023 and involved a cumulative sample size of 3205 patients, with 1604 patients in the experimental group and 1601 patients in the control group. The fundamental characteristics of the included studies are summarized in Table 1.

**TABLE 1:** Fundamental characteristics of the included studies.

Study	Inclusion criteria for subjects	Country	The number of participants (cases)		Intervention measures		Course of treatment	Outcome indicators
			T	C	T	C		
Ismael, G. 2012	1. Patients diagnosed with HER2-positive breast cancer 2. Clinical stages I to III, aged 18 years or older 3. No evidence of metastatic disease	Australia Canada Switzerland Germany Britain	297	299	A fixed-dose subcutaneous injection of 600 mg of trastuzumab is administered once every 3 weeks	Intravenous injection of trastuzumab Loading dose: 8 mg/kg Maintenance dose: 6 mg/kg Once every 3 weeks	18 cycles	①② ③
Pivot, X. 2013	1. Age ≥18 years old 2. Diagnosed as HER2-positive early breast cancer 3. Postoperative adjuvant chemotherapy has been completed 4. The eastern cooperative oncology group (ECOG) performance status score is 0 or 1	France Britain Canada Spain Denmark Germany	124	124	Patients will initially undergo four cycles of subcutaneous injections, followed by four cycles of intravenous injections. Once every 3 weeks	Patients will initially undergo four cycles of intravenous injections, followed by four cycles of subcutaneous injections. Once every 3 weeks	8 cycles	③④
Pivot, X. 2014	1. Age ≥18 years old 2. Diagnosed as HER2-positive early breast cancer 3. Postoperative adjuvant chemotherapy has been completed 4. The ECOG performance status score is 0 or 1	France Britain Canada Spain Denmark Germany	235	232	Patients will initially undergo four cycles of subcutaneous injections, followed by four cycles of intravenous injections. Once every 3 weeks	Patients will initially undergo four cycles of intravenous injections, followed by four cycles of subcutaneous injections. Once every 3 weeks	8 cycles	③④
Pivot, X. 2017	1. Age ≥18 years old 2. Diagnosed as HER2-positive breast cancer 3. The ECOG performance status score is 0 or 1 4. The baseline left ventricular ejection fraction (LVEF) was evaluated within 3 months preceding enrollment and was found to be greater than 50% 5. There was no evidence of disease progression as evaluated by clinical examination, CT scan, and bone scan within 3 weeks before inclusion	France	47	45	Patients will initially undergo four cycles of subcutaneous injections, followed by four cycles of intravenous injections. Once every 3 weeks	Patients will initially undergo four cycles of intravenous injections, followed by four cycles of subcutaneous injections. Once every 3 weeks	6 cycles	③④
Pivot 2017	1. Age ≥18 years old 2. Diagnosed as HER2-positive breast cancer 3. The ECOG performance status score is 0 or 1 4. The baseline left ventricular ejection fraction (LVEF) was evaluated within 3 months preceding enrollment and was found to be greater than 50% 5. There was no evidence of disease progression as evaluated by clinical examination, CT scan, and bone scan within 3 weeks before inclusion	America Europe Asia	244	239	Patients will initially undergo four cycles of subcutaneous injections, followed by four cycles of intravenous injections. Once every 3 weeks	Patients will initially undergo four cycles of intravenous injections, followed by four cycles of subcutaneous injections. Once every 3 weeks	8 cycles	②③
Jackisch, C. 2019	1. Age ≥18 years old 2. Diagnosed as HER2-positive breast cancer 3. Clinical stages I to III 4. No metastatic disease	Australia Canada Switzerland Germany Britain	297	299	A fixed-dose subcutaneous injection of 600 mg of trastuzumab is administered once every 3 weeks	Intravenous injection of trastuzumab Loading dose: 8 mg/kg Maintenance dose: 6 mg/kg Once every 3 weeks		①② ③⑤ ⑥
O'Shaughnessy, J. 2021	1. Age ≥18 years old 2. Diagnosed as HER2-positive early breast cancer 3. Postoperative adjuvant chemotherapy was completed, and at least two intravenous injections of trastuzumab and pertuzumab were received 4. The ECOG performance status score is 0 or 1	America Spain Finland Portugal	80	80	Patients will initially undergo four cycles of subcutaneous injections, followed by four cycles of intravenous injections. Once every 3 weeks	Patients will initially undergo four cycles of intravenous injections, followed by four cycles of subcutaneous injections. Once every 3 weeks	8 cycles	③④
Tan, A. R. 2021	1. Age ≥18 years old 2. Diagnosed as HER2-positive breast cancer 3. Surgically operable, locally advanced, or inflammatory HER2-positive (immunohistochemistry 3+ or *in situ* hybridization positive) breast cancer at stages II to IIIC, characterized by a primary tumor diameter exceeding 2 cm or the presence of lymph node involvement. 4. The ECOG performance status score is 0 or 1 5. The baseline left ventricular ejection fraction (LVEF) was evaluated within 3 months preceding enrollment and was found to be greater than 55% 6. No history of severe hepatic, renal, or hematological disorders	Europe North America Asia	248	252	A fixed-dose subcutaneous co-administration of trastuzumab and pertuzumab administered every 3 weeks	Intravenous administration of trastuzumab and pertuzumab is conducted on a three-weekly basis		①② ③⑤
Pellegrino, B. 2023	1. Age ≥18 years old 2. Patients with previously untreated, locally advanced, inflammatory, or early-stage (tumor size exceeding 2 cm or lymph node-positive) HER2-positive breast cancer who do not have metastatic disease 3. Pre-neoadjuvant therapy, tumor samples are available for examination. 4. Physical status (PS) score of 1 or lower 5. The baseline left ventricular ejection fraction (LVEF) was evaluated within 3 months preceding enrollment and was found to be greater than 55%		32	31	Subcutaneous injection of trastuzumab combined with pertuzumab and docetaxel Trastuzumab: a fixed dose of 600 mg Pertuzumab loading dose: 840 mg Maintenance dose: 420 mg Docetaxel: 75 mg/m^2^ Once every 3 weeks	Intravenous injection of trastuzumab combined with pertuzumab and docetaxel Trastuzumab: a fixed dose of 600 mg Pertuzumab loading dose: 840 mg Maintenance dose: 420 mg Docetaxel: 75 mg/m^2^ Once every 3 weeks	18 cycles	②③ ⑦

T, intervention group; C, control group.

Rate of pathological complete response (pCR) Event-free survival rateIncidence of adverse eventsPreferenceThe preoperative serum trough concentration in the eighth treatment cycleThe overall survival (OS) was followed up for six yearsClinical objective response rate.

### 3.3 Assessment outcomes regarding the quality of literature

Among the nine included studies, all adopted randomization and were assessed as having “low risk” of bias. Two studies (Jackisch et al., 2019; Tan et al., 2021) explicitly mentioned allocation concealment and were also rated as “low risk”; however, the remaining seven studies did not report on allocation concealment, leading to an “unclear risk” rating. Regarding blinding, four studies (Pivot et al., 2013; Pivot et al., 2014; Pivot et al., 2017a; Pivot et al., 2017b) demonstrated minimal subjective index bias and were rated as “low risk”. One study (Ismael et al., 2012) did not specify whether blinding was implemented for participants and researchers, resulting in an “unclear risk” rating. The remaining studies failed to adequately implement blinding and were therefore rated as “high risk”. All included studies provided complete and reliable evaluation index data, with no evidence of selective reporting, and were thus rated as “low risk”. No other biases were identified in any of the studies, which were consequently rated as “low risk”. The risk of bias graph was generated using Review Manager 5.4 software, and the specific risk of bias for each study is illustrated in Supplementary Figure 1.

### 3.4 Meta-analysis results

#### 3.4.1 Pathological complete response rate

**FIGURE 2:**
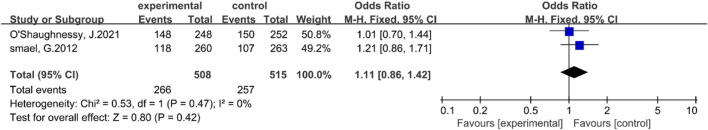
Forest plot of the pathological complete response rate.

A total of two studies (Ismael et al., 2012; O'Shaughnessy et al., 2021) reported the pathological complete response rates for both subcutaneous and intravenous administrations of trastuzumab. There was no statistically significant heterogeneity among the studies (P = 0.47, I^2^ = 0%). Therefore, a fixed-effect model was employed. The Meta-analysis results indicated that there was no statistically significant difference in the pathological complete response rate between the subcutaneous injection group and the intravenous injection group [OR = 1.11, 95% CI (0.86, 1.42), Z = 0.80, P = 0.42]. This suggests that subcutaneous and intravenous injections do not differ significantly in terms of pathological complete response rate, as illustrated in Figure 2.

#### 3.4.2 Event-free survival rate

**FIGURE 3:**
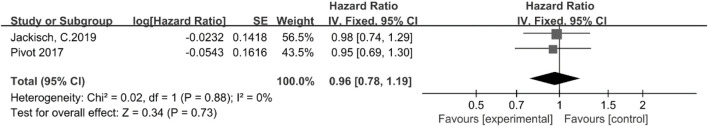
Forest plot of event-free survival time.

Two studies (Pivot et al., 2017b; Jackisch et al., 2019) reported the event-free survival period. There was no statistically significant heterogeneity between these studies (P = 0.88, I2 = 0%). Consequently, a fixed-effect model was employed for the Meta-analysis. The results indicated that there was no statistically significant difference in the event-free survival period between the subcutaneous injection group and the intravenous injection group [HR = 0.96, 95% CI (0.78, 1.19), Z = 0.34, P = 0.73]. This suggests that subcutaneous and intravenous injections do not differ significantly in terms of event-free survival, as illustrated in Figure 3.

#### 3.4.3 The incidence rate of any adverse event

**FIGURE 4:**
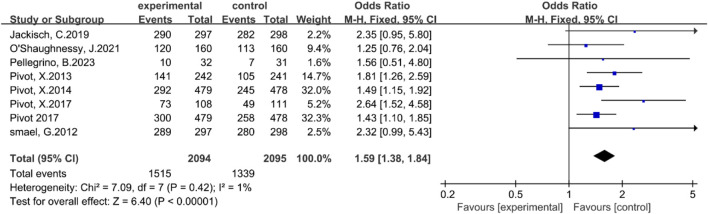
Forest plot of the incidence rate of any adverse event.

Eight studies (Ismael et al., 2012; Pivot et al., 2013; Pivot et al., 2014; Pivot et al., 2017a; Pivot et al., 2017b; Jackisch et al., 2019; O'Shaughnessy et al., 2021; Pellegrino et al., 2023) reported the incidence of any adverse events. There was no statistically significant heterogeneity among these studies (P = 0.42, I^2^ = 1%). A fixed-effect model was therefore employed. The results of the meta-analysis indicated that, compared with the intravenous injection group, the subcutaneous injection group exhibited a significantly higher incidence of any adverse events, with statistical significance [OR = 1.59, 95% CI (1.38, 1.84), Z = 6.40, P < 0.00001], as illustrated in Figure 4. Although the subcutaneous injection group experienced a higher incidence of local adverse events, the majority of these events were mild to moderate and did not substantially affect long-term patient treatment outcomes.

#### 3.4.4 The incidence rate of serious adverse events

**FIGURE 5:**
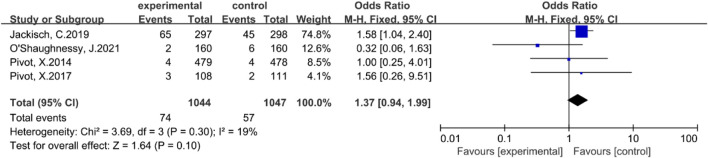
Forest plot of serious adverse events.

Four studies (Pivot et al., 2014; Pivot et al., 2017a; Jackisch et al., 2019; O'Shaughnessy et al., 2021) reported the incidence of serious adverse events. There was no statistically significant heterogeneity among these studies (P = 0.30, I2 = 19%). Consequently, a fixed-effect model was employed. The results of the meta-analysis indicated that there was no statistically significant difference in the incidence of serious adverse events between the subcutaneous injection group and the intravenous injection group [OR = 1.37, 95% CI (0.94, 1.99), Z = 1.64, P = 0.10], as illustrated in Figure 5.

#### 3.4.5 Preference

**FIGURE 6:**
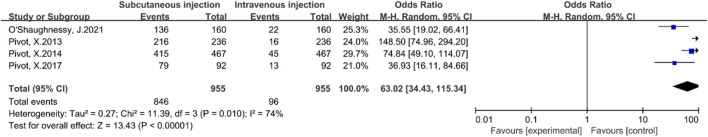
Forest plot of preference.

Four studies (Pivot et al., 2013; Pivot et al., 2014; Pivot et al., 2017a; O'Shaughnessy et al., 2021) reported patient preferences for subcutaneous *versus* intravenous injections. There was statistically significant heterogeneity among these studies (P = 0.01, I^2^ = 74%). A random-effects model was employed to account for this variability. The meta-analysis results indicated that patients significantly preferred subcutaneous injections over intravenous injections [OR = 63.02, 95% CI (34.43, 115.34), Z = 13.43, P < 0.00001], as illustrated in Figure 6. To evaluate the stability of this finding, a leave-one-out sensitivity analysis was performed. The pooled ORs ranged from 49.32 to 76.70 across iterations, consistently favoring the SC route, indicating that no single study disproportionately influenced the overall result.

### 3.5 Publication bias

Due to the limited number of studies available for each outcome (all <10), formal statistical tests for publication bias (e.g., funnel plots, Egger’s test) were not performed, as their statistical power would be insufficient to provide reliable interpretation under these conditions.

### 3.6 Assessment outcomes of the GRADE evidence grading system

The evidence level for each outcome indicator included in the meta-analysis was assessed. The results indicated that the incidence of any adverse events was supported by high-quality evidence, whereas the pathological complete response rate, event-free survival, incidence of serious adverse events were supported by moderate-quality evidence. The certainty of evidence for patient preference was rated as low, owing to substantial between-study heterogeneity (I^2^ = 74%) and the inherently subjective nature of the outcome, which may introduce variability across different study populations and settings. Refer to Supplementary Material Supplementary Table S3 for details.

### 3.7 Minimal cost analysis results

Based on the evidence from the systematic review and meta-analysis, SC and IV trastuzumab showed no significant differences in efficacy or safety among patients with HER2-positive breast cancer. Therefore, a minimal cost analysis was performed (detailed cost components are provided in the Supplementary Tables S4–S7).

#### 3.7.1 Total cost comparison

Over 18 standard treatment cycles, the total costs of different administration strategies varied substantially (Table 2). The highest total cost was observed for the IV originator product (Herceptin®, 108,026.40 CNY), while the lowest total cost was observed for the domestic biosimilar Anqutuo® (79,426.40 CNY). The SC formulation had a total cost lower than the IV originator and the domestic biosimilar Saitu®, but higher than the domestic biosimilars Hanquyou® and Anqutuo®. Compared with the IV originator, SC administration reduced total costs by approximately 7%, while the difference with domestic biosimilars ranged from 1% to 27% depending on the specific product.

**TABLE 2:** Total costs of trastuzumab by administration route over 18 cycles (CNY).

Cost component	SC trastuzumab 600 mg/vial	IV originator (Herceptin® 440 mg/vial)	IV biosimilar (Hanquyou® 150 mg/vial)	IV biosimilar (Saitu® 150 mg/vial)	IV biosimilar (Anqutuo® 150 mg/vial)
Drug acquisition	86400	93500	92800	87285	64900
Consumables	338.4	583.92	583.92	583.92	583.92
Drug management	693	783	783	783	783
Monitoring and laboratory	13165.6	13165.6	13165.6	13165.6	13165.6
Total	100597	108032.52	107332.52	101817.52	79432.52

SC, dosing was fixed at 600 mg per cycle; IV, dosing was weight-based (initial 8 mg/kg, maintenance 6 mg/kg) according to each formulation’s label and the mean patient body weight of 59 kg. Drug costs were calculated based on actual vial usage. Consumables, drug management, and monitoring/laboratory costs were accumulated over the entire treatment cycle.

### 3.8 Results of sensitivity analysis

To assess the robustness of the results, a one-way sensitivity analysis was conducted by varying key parameters, including drug price, patient body weight, consumable costs, and drug management fees, by ±20%. The analysis demonstrated that although total costs fluctuated slightly across parameter ranges, the overall cost hierarchy among regimens remained unchanged, indicating strong model robustness. Drug price and patient body weight were identified as the primary drivers of cost variation. For the subcutaneous formulation, a ±20% change in drug price resulted in approximately a 17% change in total cost, whereas the total costs of intravenous formulations (Herceptin, Hanquyou, Saitu®, and Anqutuo®) increased substantially with higher patient body weight. In contrast, variations in consumable, management, and monitoring costs exerted only minimal influence on overall expenditure. These findings highlight that drug price and dosing weight are key determinants of trastuzumab administration cost. Detailed in the comprehensive single-factor sensitivity analysis refer to Supplementary Figures 2–6.

## 4 Discussion

This meta-analysis included nine studies evaluating the efficacy, safety, and patient preference of SC *versus* IV trastuzumab in patients with HER2-positive breast cancer. The pooled results showed no statistically significant differences in pCR or EFS between the two administration routes, supporting their clinical equivalence. These findings are consistent with results from pivotal trials such as HannaH (Pivot et al., 2013), reinforcing the therapeutic interchangeability of SC and IV trastuzumab.

Regarding safety, SC administration was associated with a higher incidence of adverse events, predominantly mild-to-moderate local injection site reactions such as pain, erythema, or induration. These are characteristic of subcutaneous delivery and are typically manageable without affecting long-term treatment adherence. Importantly, no significant difference was observed in serious adverse events, confirming that the SC formulation maintains a comparable safety profile to IV administration.

Patient preference strongly favored SC administration of trastuzumab over IV administration, with a pooled odds ratio of 63.02. Considerable heterogeneity was observed among studies (I^2^ = 74%), likely reflecting differences in healthcare settings (e.g., home-based *versus* hospital-only administration), prior treatment experience, and cultural attitudes toward injections across study populations. To assess the robustness of this finding, a leave-one-out sensitivity analysis was conducted. Across all iterations, the pooled ORs ranged from 49.32 to 76.70, indicating that the observed heterogeneity did not materially affect the direction or statistical significance of the result.

The pronounced preference for SC administration appears largely attributable to its practical advantages, including markedly reduced treatment time, fewer venous punctures, and greater convenience—factors consistently valued by patients in real-world settings. These results are in line with the pivotal PrefHer study (Pivot et al., 2017a) and underscore the clinical importance of incorporating patient-centered considerations, such as comfort, treatment burden, and convenience, into therapeutic decision-making for HER2-positive breast cancer.

Importantly, most studies included in this review focused on short-to medium-term outcomes. Data on long-term OS and late-onset toxicities remain limited. A key reason for the scarcity of OS data lies in the methodological and logistical challenges of OS assessment. Detecting meaningful differences in OS typically requires very large sample sizes and extended follow-up durations often several years to capture sufficient survival events. Furthermore, when clinical efficacy is presumed equivalent, as is the case for SC and IV trastuzumab, substantial divergence in OS is neither expected nor commonly observed. As such, OS is rarely selected as a primary endpoint in comparative trials of administration routes for the same biologic agent. Among the limited evidence available, one study (Jackisch et al., 2019) reported identical OS rates of 84% in both groups, with a HR of 0.94 (95% CI: 0.61–1.45), indicating no statistically significant difference in long-term survival. These findings reinforce the therapeutic interchangeability of the two formulations and support the rationale for assessing non-survival endpoints such as patient preference, quality of life, and healthcare resource utilization. This evidence gap also underscores a major contribution of the present meta-analysis: the identification of unmet research needs that can inform future randomized controlled trial (RCT) design. Longitudinal real-world data, especially those derived from national cancer registries or electronic health records, are warranted to assess the durability and late safety profiles of SC *versus* IV trastuzumab. Such efforts are essential to ensure evidence-based decision-making over the full treatment trajectory.

Beyond clinical considerations, economic impact has become increasingly relevant in determining optimal treatment strategies. Previous reports suggest that SC trastuzumab may reduce healthcare resource utilization, including infusion time, chair time, and staffing needs (Pivot et al., 2013; Pivot et al., 2017a; Pivot et al., 2017b). For instance, the PrefHer substudy reported a reduction in chair time from approximately 90 min (IV) to 5–10 min (SC), accompanied by a 50%–60% reduction in active healthcare provider time per cycle (De Cock et al., 2016). A Spanish time-and-motion study reported active nursing time reduced from 27.2 to 13.2 min, yielding a cost saving of ∼€979 per patient over 18 cycles (Lopez-Vivanco et al., 2017). In Greece, cost-minimization analyses demonstrate lower total therapy costs with SC (e.g., €21,870 vs. €23,118) attributed mainly to reduced drug preparation and administration costs (Mylonas et al., 2017). Such consistent findings underscore the resource-efficiency of SC trastuzumab across diverse healthcare systems.

In China, both IV and SC formulations of trastuzumab are included in the NRDL. However, practical and logistical differences persist. IV formulations continue to dominate due to earlier market availability, centralized procurement practices, and formulary constraints in some tertiary hospitals. Conversely, SC administration may offer indirect cost savings by lowering outpatient infusion volumes, reducing nursing workload, and shortening patient waiting times—benefits that are particularly valuable in high-volume oncology centers. To address this evidence gap, we performed a CMA. This methodological approach is justified by the clinical equivalence between SC and IV formulations demonstrated in our meta-analysis, satisfying the fundamental CMA prerequisite of therapeutic equivalence. While comprehensive cost-effectiveness (CEA) or cost-utility analyses (CUA) would be necessary to capture indirect benefits—such as patient convenience, quality-of-life improvements, and adherence—CMA provides a rigorous, preliminary assessment of direct medical costs under the current Chinese reimbursement framework. Our findings indicate that under the current Chinese reimbursement and pricing framework, the total cost of the SC trastuzumab regimen was significantly lower than that of various IV formulations. One-way sensitivity analyses indicated that the cost ranking remained consistent across a ±20% variation in drug prices and patient body weight, suggesting the robustness of the model outcomes. Drug price and body weight were identified as the primary drivers of cost differences, highlighting the crucial influence of unit-dose cost and weight-based dosing on economic outcomes, whereas the effects of consumables and drug management fees were relatively limited. These findings underscore that further price reductions for the SC formulation or optimization of weight-based pricing mechanisms could amplify its economic advantage, thereby improving affordability and resource efficiency within the healthcare system. Against the backdrop of China’s current reimbursement policy, the wider implementation of SC trastuzumab carries substantial practical and policy significance. Its markedly reduced administration time can indirectly lower labor costs in infusion units and increase patient throughput, thereby enhancing hospital operational efficiency and optimizing resource allocation. From the payer perspective, selecting a lower-cost administration route without compromising clinical efficacy is consistent with the principles of value-based reimbursement. From the patient standpoint, SC administration minimizes discomfort associated with intravenous access, reduces waiting time, and improves treatment experience and adherence, which may in turn positively influence therapeutic outcomes. Collectively, these clinical, economic, and patient-centered advantages position SC trastuzumab as a highly competitive strategy within value-based oncology care. It should be acknowledged that the CMA framework inherently focuses on direct costs and does not quantify broader benefits of SC administration, including patient time savings, convenience, and potential improvements in adherence and quality of life. These dimensions are critical for future, more comprehensive pharmacoeconomic evaluations. Future studies should integrate real-world cost data, long-term clinical outcomes, and patient-reported quality-of-life metrics, ideally via CEA or CUA frameworks, to comprehensively assess the holistic value of SC trastuzumab and inform evidence-based reimbursement and resource allocation decisions in China.

These findings carry particular significance for regions where SC trastuzumab has only recently been introduced into clinical practice. For example, while SC trastuzumab has been approved in Europe since 2013 and in the United States since 2019, it was only approved in China in 2022 (Waller et al., 2021). In such settings, clinicians are still navigating the adoption process, and high-quality comparative evidence can support informed treatment decisions and policy development. The results of this study offer timely guidance for countries and regions seeking to transition toward more patient-centered and resource-efficient treatment models.

Nonetheless, several limitations should be acknowledged. First, heterogeneity was present in study designs, patient populations, and follow-up durations, which may influence the pooled outcomes. Second, the primary endpoints of this meta-analysis were pCR and EFS, as these were the outcomes most consistently reported across the included trials. Long-term survival data were notably limited: only one study reported OS; therefore, formal meta-analysis of OS was not feasible, and the precision of the pooled EFS estimate may be constrained by the small number of contributing studies. Furthermore, heterogeneity and publication bias for survival endpoints could not be reliably assessed due to insufficient study numbers. These limitations indicate that while current evidence supports comparable short-to medium-term efficacy between SC and IV trastuzumab, conclusions regarding long-term survival equivalence remain preliminary and should be interpreted with caution. Third, patient preference for administration route was derived from subjective surveys and may be influenced by cultural context, healthcare system, and prior treatment experience. Considerable heterogeneity among studies and the absence of subgroup or sensitivity analyses may limit the certainty and generalizability of the evidence. Fourth, a cost-minimization analysis was conducted based on presumed clinical equivalence between SC and IV formulations. CMA focuses on direct medical costs and may not capture broader aspects such as patient time, convenience, adherence, quality of life, or indirect costs. These factors could provide additional advantages of SC administration. Future cost-effectiveness or cost-utility studies would help more comprehensively assess the value of SC trastuzumab in China. Finally, formal assessment of publication bias (e.g., funnel plots) was not feasible due to the limited number of included studies (<10). Notably, several included trials were industry-sponsored, introducing the potential for sponsorship bias. Although our risk of bias assessment identified no overt methodological issues, the possibility of unpublished negative findings cannot be entirely excluded. Despite our broad search strategy to mitigate these risks, findings should be interpreted with this context in mind.

Future studies should aim to: (1) conduct randomized controlled trials with standardized methodology across diverse regions; (2) include long-term follow-up to assess durable outcomes; (3) performing comprehensive cost-effectiveness or cost-utility analyses, integrating real-world clinical outcomes and patient-reported quality-of-life measures to fully quantify treatment value; (4) explore the comparative value of SC trastuzumab in specific subgroups, such as elderly patients, those with limited venous access, or populations with low treatment adherence.

## 5 Conclusion

SC trastuzumab shows comparable efficacy and safety to IV administration in HER2-positive breast cancer, with no significant differences in pathological complete response or event-free survival. Injection site reactions are more frequent but mild and transient. Given clinical equivalence, patient preference driven by reduced administration time and convenience is a key factor. Subcutaneous delivery may improve resource utilization, especially in high-volume or resource-constrained settings. Cost-minimization analysis based on the meta-analysis results indicates that SC administration has a potential cost advantage under the current reimbursement and drug pricing system. Taken together, the clinical, patient-centered, and economic benefits suggest that SC trastuzumab has strong potential for broader adoption, providing valuable guidance for future clinical practice and health policy decision-making.

## Data Availability

The original contributions presented in the study are included in the article/Supplementary Material, further inquiries can be directed to the corresponding authors.
