# Supplementary material for: Subcutaneous versus intravenous trastuzumab for HER2-positive breast cancer: a global systematic review and meta-analysis with a cost-minimization analysis from the Chinese healthcare system perspective
Source: Front Pharmacol. 2026 Jan 7;16:1730175. doi: 10.3389/fphar.2025.1730175 (PMC12819671; doi:10.3389/fphar.2025.1730175)
Supplement: Supplementary file 1 [file Supplementaryfile1.docx]

**Supplementary** **Material**

[Table S1 The search strategy of database search 2](#_Toc215921828)

[Table S2 List of Excluded Studies with Reasons 12](#_Toc215921829)

[Table S3. GRADE Quality Assessment Table 48](#_Toc215921830)

[Table S4.Total drug costs of different trastuzumab formulations over 18 treatment cycles(CNY) 49](#_Toc215921831)

[Table S5. Estimation of the Consumable Costs for Different Dosage Forms of Trastuzumab(CNY) 50](#_Toc215921832)

[Table S6. Administration and Service Costs for Subcutaneous and Intravenous Trastuzumab 51](#_Toc215921833)

[Table S7. Monitoring and Laboratory Costs for Trastuzumab Treatment 52](#_Toc215921834)

[Figure 1. Risk of Bias Assessment Diagram for Randomized Controlled Trials 53](#_Toc215921835)

[Figure 2. One-Way Sensitivity Analysis: Subcutaneous Trastuzumab 54](#_Toc215921836)

[Figure 3. One-Way Sensitivity Analysis: Intravenous Originator Trastuzumab (Herceptin®) 54](#_Toc215921837)

[Figure 4. One-Way Sensitivity Analysis: Intravenous Originator Trastuzumab (Hanquyou®) 55](#_Toc215921838)

[Figure 5. One-Way Sensitivity Analysis: Intravenous Originator Trastuzumab(Saitu®) 55](#_Toc215921839)

[Figure 6. One-Way Sensitivity Analysis: Intravenous Originator Trastuzumab (Anqutuo®) 56](#_Toc215921840)

[PRISMA checklist 57](#_Toc215921841)

# Table S1 The search strategy of database search

| **PubMed** | | |
| --- | --- | --- |
| ID | Search Terms | Results |
| #1 | "Breast Neoplasms"[Mesh] | 362925 |
| #2 | (((((((((((((Breast Neoplasms[Title/Abstract]) OR (Breast Neoplasm[Title/Abstract])) OR (Neoplasm, Breast[Title/Abstract])) OR (Neoplasms, Breast[Title/Abstract])) OR (Breast Tumors[Title/Abstract])) OR (Breast Tumor[Title/Abstract])) OR (Tumor, Breast[Title/Abstract])) OR (Tumors, Breast[Title/Abstract])) OR (Breast Cancer[Title/Abstract])) OR (Cancer, Breast[Title/Abstract])) OR (Cancer of Breast[Title/Abstract])) OR (Cancer of the Breast[Title/Abstract])) OR (Malignant Neoplasm of Breast[Title/Abstract])) OR (Breast Malignant Neoplasm[Title/Abstract]) | 386055 |
| #3 | ("Breast Neoplasms"[Mesh]) OR ((((((((((((((Breast Neoplasms[Title/Abstract]) OR (Breast Neoplasm[Title/Abstract])) OR (Neoplasm, Breast[Title/Abstract])) OR (Neoplasms, Breast[Title/Abstract])) OR (Breast Tumors[Title/Abstract])) OR (Breast Tumor[Title/Abstract])) OR (Tumor, Breast[Title/Abstract])) OR (Tumors, Breast[Title/Abstract])) OR (Breast Cancer[Title/Abstract])) OR (Cancer, Breast[Title/Abstract])) OR (Cancer of Breast[Title/Abstract])) OR (Cancer of the Breast[Title/Abstract])) OR (Malignant Neoplasm of Breast[Title/Abstract])) OR (Breast Malignant Neoplasm[Title/Abstract])) | 481181 |
| #4 | "Receptor, ErbB-2"[Mesh] | 31266 |
| #5 | (((((((((((((((ErbB-2 Receptor[Title/Abstract]) OR (Erb-b2 Receptor Tyrosine Kinases[Title/Abstract])) OR (HER-2 Proto-Oncogene Protein[Title/Abstract])) OR (HER 2 Proto Oncogene Protein[Title/Abstract])) OR (Proto-Oncogene Protein, HER-2[Title/Abstract])) OR (Proto-Oncogene Protein HER-2[Title/Abstract])) OR (Oncogene Protein HER-2[Title/Abstract])) OR (Proto-Oncogene Protein p185(neu[Title/Abstract]))) OR (Proto-Oncogene Proteins c-erbB-2[Title/Abstract])) OR (c-erbB-2 Protein[Title/Abstract])) OR (c erbB 2 Protein[Title/Abstract])) OR (erbB-2 Proto-Oncogene Protein[Title/Abstract])) OR (erbB 2 Proto Oncogene Protein[Title/Abstract])) OR (erbB-2 Receptor Protein-Tyrosine Kinase[Title/Abstract])) OR (neu Proto-Oncogene Protein[Title/Abstract])) OR (neu Proto Oncogene Protein[Title/Abstract]) | 716 |
| #6 | ("Receptor, ErbB-2"[Mesh]) OR ((((((((((((((((ErbB-2 Receptor[Title/Abstract]) OR (Erb-b2 Receptor Tyrosine Kinases[Title/Abstract])) OR (HER-2 Proto-Oncogene Protein[Title/Abstract])) OR (HER 2 Proto Oncogene Protein[Title/Abstract])) OR (Proto-Oncogene Protein, HER-2[Title/Abstract])) OR (Proto-Oncogene Protein HER-2[Title/Abstract])) OR (Oncogene Protein HER-2[Title/Abstract])) OR (Proto-Oncogene Protein p185(neu[Title/Abstract]))) OR (Proto-Oncogene Proteins c-erbB-2[Title/Abstract])) OR (c-erbB-2 Protein[Title/Abstract])) OR (c erbB 2 Protein[Title/Abstract])) OR (erbB-2 Proto-Oncogene Protein[Title/Abstract])) OR (erbB 2 Proto Oncogene Protein[Title/Abstract])) OR (erbB-2 Receptor Protein-Tyrosine Kinase[Title/Abstract])) OR (neu Proto-Oncogene Protein[Title/Abstract])) OR (neu Proto Oncogene Protein[Title/Abstract])) | 31401 |
| #7 | (("Breast Neoplasms"[Mesh]) OR ((((((((((((((Breast Neoplasms[Title/Abstract]) OR (Breast Neoplasm[Title/Abstract])) OR (Neoplasm, Breast[Title/Abstract])) OR (Neoplasms, Breast[Title/Abstract])) OR (Breast Tumors[Title/Abstract])) OR (Breast Tumor[Title/Abstract])) OR (Tumor, Breast[Title/Abstract])) OR (Tumors, Breast[Title/Abstract])) OR (Breast Cancer[Title/Abstract])) OR (Cancer, Breast[Title/Abstract])) OR (Cancer of Breast[Title/Abstract])) OR (Cancer of the Breast[Title/Abstract])) OR (Malignant Neoplasm of Breast[Title/Abstract])) OR (Breast Malignant Neoplasm[Title/Abstract]))) AND (("Receptor, ErbB-2"[Mesh]) OR ((((((((((((((((ErbB-2 Receptor[Title/Abstract]) OR (Erb-b2 Receptor Tyrosine Kinases[Title/Abstract])) OR (HER-2 Proto-Oncogene Protein[Title/Abstract])) OR (HER 2 Proto Oncogene Protein[Title/Abstract])) OR (Proto-Oncogene Protein, HER-2[Title/Abstract])) OR (Proto-Oncogene Protein HER-2[Title/Abstract])) OR (Oncogene Protein HER-2[Title/Abstract])) OR (Proto-Oncogene Protein p185(neu[Title/Abstract]))) OR (Proto-Oncogene Proteins c-erbB-2[Title/Abstract])) OR (c-erbB-2 Protein[Title/Abstract])) OR (c erbB 2 Protein[Title/Abstract])) OR (erbB-2 Proto-Oncogene Protein[Title/Abstract])) OR (erbB 2 Proto Oncogene Protein[Title/Abstract])) OR (erbB-2 Receptor Protein-Tyrosine Kinase[Title/Abstract])) OR (neu Proto-Oncogene Protein[Title/Abstract])) OR (neu Proto Oncogene Protein[Title/Abstract]))) | 22118 |
| #8 | "Trastuzumab"[Mesh] | 9616 |
| #9 | ((((((Trastuzumab[Title/Abstract]) OR (Trastuzumab beta[Title/Abstract])) OR (beta, Trastuzumab[Title/Abstract])) OR (Herceptin[Title/Abstract])) OR (Trazimera[Title/Abstract])) OR (Trastuzumab-qyyp[Title/Abstract])) OR (Trastuzumab qyyp[Title/Abstract]) | 15166 |
| #10 | ("Trastuzumab"[Mesh]) OR (((((((Trastuzumab[Title/Abstract]) OR (Trastuzumab beta[Title/Abstract])) OR (beta, Trastuzumab[Title/Abstract])) OR (Herceptin[Title/Abstract])) OR (Trazimera[Title/Abstract])) OR (Trastuzumab-qyyp[Title/Abstract])) OR (Trastuzumab qyyp[Title/Abstract])) | 16557 |
| #11 | "Infusions, Intravenous"[Mesh] | 57696 |
| #12 | ((((((Trastuzumab[Title/Abstract]) OR (Trastuzumab beta[Title/Abstract])) OR (beta, Trastuzumab[Title/Abstract])) OR (Herceptin[Title/Abstract])) OR (Trazimera[Title/Abstract])) OR (Trastuzumab-qyyp[Title/Abstract])) OR (Trastuzumab qyyp[Title/Abstract]) | 15166 |
| #13 | ("Trastuzumab"[Mesh]) OR (((((((Trastuzumab[Title/Abstract]) OR (Trastuzumab beta[Title/Abstract])) OR (beta, Trastuzumab[Title/Abstract])) OR (Herceptin[Title/Abstract])) OR (Trazimera[Title/Abstract])) OR (Trastuzumab-qyyp[Title/Abstract])) OR (Trastuzumab qyyp[Title/Abstract])) | 16557 |
| #14 | "Infusions, Intravenous"[Mesh] | 57696 |
| #15 | (((((((((Intravenous[Title/Abstract]) OR (Intravenous Infusions[Title/Abstract])) OR (Infusion, Intravenous[Title/Abstract])) OR (Intravenous Infusion[Title/Abstract])) OR (Intravenous Drip[Title/Abstract])) OR (Drip, Intravenous[Title/Abstract])) OR (Drip Infusions[Title/Abstract])) OR (Drip Infusion[Title/Abstract])) OR (Infusion, Drip[Title/Abstract])) OR (Infusions, Drip[Title/Abstract]) | 340557 |
| #16 | ("Infusions, Intravenous"[Mesh]) OR ((((((((((Intravenous[Title/Abstract]) OR (Intravenous Infusions[Title/Abstract])) OR (Infusion, Intravenous[Title/Abstract])) OR (Intravenous Infusion[Title/Abstract])) OR (Intravenous Drip[Title/Abstract])) OR (Drip, Intravenous[Title/Abstract])) OR (Drip Infusions[Title/Abstract])) OR (Drip Infusion[Title/Abstract])) OR (Infusion, Drip[Title/Abstract])) OR (Infusions, Drip[Title/Abstract])) | 370834 |
| #17 | (("Trastuzumab"[Mesh]) OR (((((((Trastuzumab[Title/Abstract]) OR (Trastuzumab beta[Title/Abstract])) OR (beta, Trastuzumab[Title/Abstract])) OR (Herceptin[Title/Abstract])) OR (Trazimera[Title/Abstract])) OR (Trastuzumab-qyyp[Title/Abstract])) OR (Trastuzumab qyyp[Title/Abstract]))) AND (("Infusions, Intravenous"[Mesh]) OR ((((((((((Intravenous[Title/Abstract]) OR (Intravenous Infusions[Title/Abstract])) OR (Infusion, Intravenous[Title/Abstract])) OR (Intravenous Infusion[Title/Abstract])) OR (Intravenous Drip[Title/Abstract])) OR (Drip, Intravenous[Title/Abstract])) OR (Drip Infusions[Title/Abstract])) OR (Drip Infusion[Title/Abstract])) OR (Infusion, Drip[Title/Abstract])) OR (Infusions, Drip[Title/Abstract]))) | 513 |
| #18 | "Infusions, Subcutaneous"[Mesh] | 1427 |
| #19 | (((Subcutaneous[Title/Abstract]) OR (Infusion, Subcutaneous[Title/Abstract])) OR (Subcutaneous Infusion[Title/Abstract])) OR (Subcutaneous Infusions[Title/Abstract]) | 162536 |
| #20 | ("Infusions, Subcutaneous"[Mesh]) OR ((((Subcutaneous[Title/Abstract]) OR (Infusion, Subcutaneous[Title/Abstract])) OR (Subcutaneous Infusion[Title/Abstract])) OR (Subcutaneous Infusions[Title/Abstract])) | 162891 |
| #21 | (("Trastuzumab"[Mesh]) OR (((((((Trastuzumab[Title/Abstract]) OR (Trastuzumab beta[Title/Abstract])) OR (beta, Trastuzumab[Title/Abstract])) OR (Herceptin[Title/Abstract])) OR (Trazimera[Title/Abstract])) OR (Trastuzumab-qyyp[Title/Abstract])) OR (Trastuzumab qyyp[Title/Abstract]))) AND (("Infusions, Subcutaneous"[Mesh]) OR ((((Subcutaneous[Title/Abstract]) OR (Infusion, Subcutaneous[Title/Abstract])) OR (Subcutaneous Infusion[Title/Abstract])) OR (Subcutaneous Infusions[Title/Abstract]))) | 288 |
| #22 | (((("Breast Neoplasms"[Mesh]) OR ((((((((((((((Breast Neoplasms[Title/Abstract]) OR (Breast Neoplasm[Title/Abstract])) OR (Neoplasm, Breast[Title/Abstract])) OR (Neoplasms, Breast[Title/Abstract])) OR (Breast Tumors[Title/Abstract])) OR (Breast Tumor[Title/Abstract])) OR (Tumor, Breast[Title/Abstract])) OR (Tumors, Breast[Title/Abstract])) OR (Breast Cancer[Title/Abstract])) OR (Cancer, Breast[Title/Abstract])) OR (Cancer of Breast[Title/Abstract])) OR (Cancer of the Breast[Title/Abstract])) OR (Malignant Neoplasm of Breast[Title/Abstract])) OR (Breast Malignant Neoplasm[Title/Abstract]))) AND (("Receptor, ErbB-2"[Mesh]) OR ((((((((((((((((ErbB-2 Receptor[Title/Abstract]) OR (Erb-b2 Receptor Tyrosine Kinases[Title/Abstract])) OR (HER-2 Proto-Oncogene Protein[Title/Abstract])) OR (HER 2 Proto Oncogene Protein[Title/Abstract])) OR (Proto-Oncogene Protein, HER-2[Title/Abstract])) OR (Proto-Oncogene Protein HER-2[Title/Abstract])) OR (Oncogene Protein HER-2[Title/Abstract])) OR (Proto-Oncogene Protein p185(neu[Title/Abstract]))) OR (Proto-Oncogene Proteins c-erbB-2[Title/Abstract])) OR (c-erbB-2 Protein[Title/Abstract])) OR (c erbB 2 Protein[Title/Abstract])) OR (erbB-2 Proto-Oncogene Protein[Title/Abstract])) OR (erbB 2 Proto Oncogene Protein[Title/Abstract])) OR (erbB-2 Receptor Protein-Tyrosine Kinase[Title/Abstract])) OR (neu Proto-Oncogene Protein[Title/Abstract])) OR (neu Proto Oncogene Protein[Title/Abstract])))) AND ((("Trastuzumab"[Mesh]) OR (((((((Trastuzumab[Title/Abstract]) OR (Trastuzumab beta[Title/Abstract])) OR (beta, Trastuzumab[Title/Abstract])) OR (Herceptin[Title/Abstract])) OR (Trazimera[Title/Abstract])) OR (Trastuzumab-qyyp[Title/Abstract])) OR (Trastuzumab qyyp[Title/Abstract]))) AND (("Infusions, Intravenous"[Mesh]) OR ((((((((((Intravenous[Title/Abstract]) OR (Intravenous Infusions[Title/Abstract])) OR (Infusion, Intravenous[Title/Abstract])) OR (Intravenous Infusion[Title/Abstract])) OR (Intravenous Drip[Title/Abstract])) OR (Drip, Intravenous[Title/Abstract])) OR (Drip Infusions[Title/Abstract])) OR (Drip Infusion[Title/Abstract])) OR (Infusion, Drip[Title/Abstract])) OR (Infusions, Drip[Title/Abstract]))))) AND ((("Trastuzumab"[Mesh]) OR (((((((Trastuzumab[Title/Abstract]) OR (Trastuzumab beta[Title/Abstract])) OR (beta, Trastuzumab[Title/Abstract])) OR (Herceptin[Title/Abstract])) OR (Trazimera[Title/Abstract])) OR (Trastuzumab-qyyp[Title/Abstract])) OR (Trastuzumab qyyp[Title/Abstract]))) AND (("Infusions, Subcutaneous"[Mesh]) OR ((((Subcutaneous[Title/Abstract]) OR (Infusion, Subcutaneous[Title/Abstract])) OR (Subcutaneous Infusion[Title/Abstract])) OR (Subcutaneous Infusions[Title/Abstract])))) | 63 |
| **Web of Science** | | |
| ID | Search Terms | Results |
| #1 | HER2-postive breast cancer (Topic) OR HER2 breast cancer (Topic) and Preprint Citation Index (Exclude – Database) | 64377 |
| #2 | Intravenous (Topic) OR IV (Topic) OR IV administration (Topic) and Preprint Citation Index (Exclude – Database) | 2135064 |
| #3 | trastuzumab (Topic) OR Herceptin (Topic) and Preprint Citation Index (Exclude – Database) | 38661 |
| #4 | #2 AND #3 and Preprint Citation Index (Exclude – Database) | 2007 |
| #5 | Subcutaneous (Topic) OR Subcutaneous Infusion (Topic) OR Subcutaneous injection (Topic) and Preprint Citation Index (Exclude – Database) | 304833 |
| #6 | #3 AND #5 and Preprint Citation Index (Exclude – Database) | 710 |
| #7 | #1 AND #4 AND #6 and Preprint Citation Index (Exclude – Database) | 175 |
| **Embase** | | |
| ID | Search Terms | Results |
| #1 | 'human epidermal growth factor receptor 2 positive breast cancer'/exp | 14659 |
| #2 | ('human epidermal growth factor receptor 2 positive breast cancer':ab,ti OR 'human epidermal growth factor receptor 2 positive breast carcinoma':ab,ti OR 'her2-positive breast carcinoma':ab,ti OR 'her2-positive early breast cancer':ab,ti OR 'her2+ early breast cancer':ab,ti OR 'her2-positive breast cancer':ab,ti OR 'her2+ breast cancer':ab,ti OR 'her2+ breast carcinoma':ab,ti OR 'her+ breast cancer':ab,ti OR 'epidermal growth factor receptor 2 positive breast carcinoma':ab,ti) AND 'epidermal growth factor receptor 2 positive breast cancer':ab,ti | 349 |
| #3 | #1 OR #2 | 14796 |
| #4 | 'trastuzumab'/exp | 54758 |
| #5 | trastuzumab:ab,ti OR zedora:ab,ti OR vivitra:ab,ti OR ub921:ab,ti OR tx05:ab,ti OR tuznue:ab,ti OR trazimera:ab,ti OR 'trastuzumab strf':ab,ti OR 'trastuzumab qyyp':ab,ti OR 'trastuzumab pkrb':ab,ti OR 'trastuzumab dttb':ab,ti OR 'trastuzumab anns':ab,ti OR 'trastuzumab beta':ab,ti | 26538 |
| #6 | #4 OR #5 | 56777 |
| #7 | 'intravenous drug administration'/exp | 424986 |
| #8 | 'venous transfusion':ab,ti OR 'venous injection':ab,ti OR 'venous infusion':ab,ti OR 'venous drip':ab,ti OR 'intravenous drug administration':ab,ti OR 'vein infusion':ab,ti OR 'iv transfusion':ab,ti OR 'iv medication':ab,ti OR 'iv injection':ab,ti OR 'iv infusion':ab,ti OR 'iv fluid administration':ab,ti OR 'iv drug therapy':ab,ti OR 'iv drug injection':ab,ti OR 'iv drug delivery':ab,ti OR 'iv drug administration':ab,ti OR 'iv administration':ab,ti OR 'intravenous transfusion':ab,ti OR 'intravenous therapy':ab,ti OR 'intravenous medication':ab,ti OR 'intravenous injections':ab,ti OR 'intravenous injection':ab,ti OR 'intravenous infusions':ab,ti OR 'intravenous infusion':ab,ti | 107895 |
| #9 | #7 OR #8 | 485788 |
| #10 | #6 AND #9 | 694 |
| #11 | 'subcutaneous drug administration'/exp | 100346 |
| #12 | 'subcutaneous drug administration':ab,ti OR 'subcutaneous injections':ab,ti OR 'subcutaneous injection':ab,ti OR 'subcutaneous infusions':ab,ti OR 'subcutaneous infusion':ab,ti OR 'subcutaneous drug injection':ab,ti OR 'subcutaneous dose':ab,ti OR 'subcutaneous dosage':ab,ti OR 'subcutaneous application':ab,ti OR 'subcutaneous administration':ab,ti OR 'injections, subcutaneous':ab,ti OR 'infusions, subcutaneous':ab,ti OR 'infusion, subcutaneous':ab,ti OR 'drug administration, subcutaneous':ab,ti | 45417 |
| #13 | #11 OR #12 | 132880 |
| #14 | #6 AND #13 | 192 |
| #15 | #3 AND #10 AND #14 | 53 |
| **Cochrane Library** | | |
| ID | Search Terms | Results |
| #1 | MeSH descriptor: [Breast Neoplasms] explode all trees | 20844 |
| #2 | (Breast Carcinomas):ti,ab,kw OR (Neoplasm, Human Mammary):ti,ab,kw OR (Cancer of Breast):ti,ab,kw OR (Cancer of Breast):ti,ab,kw AND (Cancer of Breast):ti,ab,kw | 45798 |
| #3 | (Cancers, Mammary):ti,ab,kw OR (Breast Malignant Neoplasms):ti,ab,kw OR (Cancer of the Breast):ti,ab,kw OR (Malignant Neoplasm of Breast):ti,ab,kw OR (Malignant Tumor of Breast):ti,ab,kw | 42981 |
| #4 | (Mammary Cancer):ti,ab,kw OR (Breast Neoplasm):ti,ab,kw OR (Tumor, Breast):ti,ab,kw OR (Neoplasm, Breast):ti,ab,kw | 17018 |
| #5 | #1 OR #2 OR #3 OR #4 | 48330 |
| #6 | MeSH descriptor: [Receptor, ErbB-2] explode all trees | 1713 |
| #7 | (Receptor, ErbB-2):ti,ab,kw OR (neu Proto-Oncogene Protein):ti,ab,kw OR (Metastatic Lymph Node Gene 19 Protein):ti,ab,kw OR (erbB-2 Proto-Oncogene Protein):ti,ab,kw OR (erbB-2 Receptors):ti,ab,kw | 1769 |
| #8 | (ErbB-2 Receptor):ti,ab,kw OR (p185erbB2 Protein):ti,ab,kw OR (CD340 Antigens):ti,ab,kw OR (Receptor Protein Tyrosine Kinase):ti,ab,kw OR (Tyrosine Kinase type Cell Surface Receptor HER2):ti,ab,kw | 2480 |
| #9 | (Oncogene Protein HER-2):ti,ab,kw OR (Tyrosine Kinase-type Cell Surface Receptor HER2):ti,ab,kw OR (HER 2 Proto Oncogene Protein):ti,ab,kw OR (HER-2 Proto-Oncogene Protein):ti,ab,kw OR (Proto-Oncogene Protein, HER-2):ti,ab,kw | 15 |
| #10 | #6 OR #7 OR #8 OR #9 | 2495 |
| #11 | #5 AND #10 | 1650 |
| #12 | MeSH descriptor: [Trastuzumab] explode all trees | 1264 |
| #13 | (Trastuzumab):ti,ab,kw OR (Trastuzumab beta):ti,ab,kw OR (beta, Trastuzumab):ti,ab,kw OR (Trastuzumab-qyyp):ti,ab,kw OR (Trazimera):ti,ab,kw | 3842 |
| #14 | (Trastuzumab qyyp):ti,ab,kw OR (Herceptin):ti,ab,kw | 558 |
| #15 | #12 OR #13 OR #14 | 3920 |
| #16 | MeSH descriptor: [Infusions, Intravenous] explode all trees | 12440 |
| #17 | (Intravenous Infusions):ti,ab,kw OR (Infusion, Intravenous):ti,ab,kw OR (Intravenous Infusion):ti,ab,kw OR (Drip, Intravenous):ti,ab,kw OR (Infusions, Drip):ti,ab,kw | 40111 |
| #18 | (Drip Infusions):ti,ab,kw OR (Infusion, Drip):ti,ab,kw OR (Drip Infusion):ti,ab,kw OR (Intravenous Drip):ti,ab,kw | 1221 |
| #19 | #16 OR #17 OR #18 | 40245 |
| #20 | #15 AND #19 | 165 |
| #21 | MeSH descriptor: [Infusions, Intravenous] explode all trees | 12440 |
| #22 | (Intravenous Infusions):ti,ab,kw OR (Infusion, Intravenous):ti,ab,kw OR (Intravenous Infusion):ti,ab,kw OR (Drip, Intravenous):ti,ab,kw OR (Infusions, Drip):ti,ab,kw | 40111 |
| #23 | (Drip Infusions):ti,ab,kw OR (Infusion, Drip):ti,ab,kw OR (Drip Infusion):ti,ab,kw OR (Intravenous Drip):ti,ab,kw | 1221 |
| #24 | #21 OR #22 OR #23 | 40425 |
| #25 | #15 AND #24 | 165 |
| #26 | #11 AND #20 AND #25 | 37 |

# Table S2 List of Excluded Studies with Reasons

|  | **Citation** | **Reason** |  |
| --- | --- | --- | --- |
| [1] | Monoclonal antibodies as adjuvant breast cancer therapy. Tumor cells in continuing sleep? [J]. MMW Fortschritte der Medizin, 2001, 143(44): 56. | Irrelevant study |  |
| [2] | Subcutaneous trastuzumab (Herceptin). Ready to use, but more serious adverse effects than intravenous (IV) trastuzumab [J]. Prescrire international, 2015, 24(159): 93. | Irrelevant study |  |
| [3] | Aguiar-Ibanez R, Fotheringham I, Mittal L, Sillah A, Pathak S. - Differences Between Intravenous and Subcutaneous Modes of Administration in Oncology from the Patient, Healthcare Provider, and Healthcare System Perspectives: A Systematic Review [J]. 2024, - 41(- 12): - 4417. | The research type does not match. |  |
| [4] | Ahn J S, Jackisch C, Hegg R, Stroyakovskiy D, Melichar B, Chen S C, et al. - Phase III HannaH study of subcutaneous or intravenous trastuzumab for HER2-positive early breast cancer: Exploratory subgroup analyses of pathological complete response and 3-year event-free survival by body weight and anti-drug antibody status [J]. 2015, - 26: - 17. | The research design is inconsistent. |  |
| [5] | Alexander M, Jachno K, Phillips K A, Seymour J F, Slavin M A, Cheung A, et al. Infective complications in cancer patients treated with subcutaneous versus intravenous trastuzumab and rituximab: An individual patient data meta-analysis [J]. Journal of Oncology Pharmacy Practice, 2024, 30(4): 642-660. | The research type does not match. |  |
| [6] | Allmendinger A. Opportunities in an Evolving Pharmaceutical Development Landscape: Product Differentiation of Biopharmaceutical Drug Products [J]. Pharmaceutical Research, 2021, 38(5): 739-757. | Irrelevant study |  |
| [7] | Altini M, Gentili N, Balzi W, Musuraca G, Maltoni R, Masini C, et al. The challenge of sustainability in healthcare systems: economic and organizational impact of subcutaneous formulations for rituximab and trastuzumab in onco-hematology [J]. Expert review of pharmacoeconomics & outcomes research, 2021, 21(3): 503-509. | Irrelevant study |  |
| [8] | Arora S, Gogia D A, Deo S, Sharma D, Mathur S R. - Neoadjuvant pertuzumab plus trastuzumab in combination with anthracycline- free chemotherapy regimen in patients with HER2 positive breast cancer-Real-world data from a single center in India [J]. 2021, - 29: - 100483. | The research design is inconsistent. |  |
| [9] | Baselga J, Bradbury I, Eidtmann H, Di Cosimo S, de Azambuja E, Aura C, et al. Lapatinib with trastuzumab for HER2-positive early breast cancer (NeoALTTO): a randomised, open-label, multicentre, phase 3 trial [J]. Lancet (London, England), 2012, 379(9816): 633‐640. | The research design is inconsistent. |  |
| [10] | Belleudi V, Rosa A C, Musicco F, Marchetti P, Martini N, Andriani A, et al. - [Appropriate use of trastuzumab in Lazio Region: therapeutic scenarios and estimation of possible savings for the Regional Health Service.] [J]. 2019, - 110(- 12): - 614. | Irrelevant study |  |
| [11] | Bellone M, Pradelli L, Sanfilippo A, Caputo A, Manevy M, Zerilli A. POSC113 Fixed-Dose Combination of Pertuzumab and Trastuzumab for Subcutaneous Injection in the Treatment of HER2-Positive Breast Cancer (HER2+ BC) Patients in Italy: A Budget Impact Analysis [J]. Value in Health, 2022, 25(1): S109. | The research design is inconsistent. |  |
| [12] | Bittner B, Richter W, Schmidt J. - Subcutaneous Administration of Biotherapeutics: An Overview of Current Challenges and Opportunities [J]. 2018, - 32(- 5): - 440. | Irrelevant study |  |
| [13] | Bustamante G, Wang B, DeLuna F, Sun L, Ye J Y. - In-vivo fluorescence detection of breast cancer growth factor receptors by fiber-optic probe [J]. 2018, - 10489. | Irrelevant study |  |
| [14] | Calleja T, Galve-Calvo E, Gómez Agudo E, Llaguno J L, Martin Rufo M, Arroyo I, et al. EE528 Analysis of Non-Pharmacological Cost Savings Derived from the Use of Fixed-Dose Combination of Pertuzumab and Trastuzumab for Subcutaneous Injection in Patients with HER2-Positive Breast Cancer in Spain [J]. Value in Health, 2023, 26(12): S153. | The research design is inconsistent |  |
| [15] | Castillo-Fernandez O, Cabreja A, Arauz E, Bellido D, Lim M, Lopez R, et al. - Do patients and nurses outside clinical trial prefer subcutaneous trastuzumab over conventional intravenous infusion? Instituto Oncologico Nacional experience [J]. 2017, - 77. | Irrelevant study |  |
| [16] | Castillo-Fernandez O, Castillero F, Lim M, Martin C, Montano L, Benitez S, et al. - Effectiveness of subcutaneous trastuzumab and intravenous pertuzumab as neoadjuvant dual blockade for locally advanced HER2+breast cancer: Real world evidence from Instituto Oncologico Nacional, Panama City, Panama [J]. 2020, - 80(- 4). | The research design is inconsistent |  |
| [17] | Chan L J, Bulitta J B, Ascher D B, Haynes J M, McLeod V M, Porter C J H, et al. - PEGylation Does Not Significantly Change the Initial Intravenous or Subcutaneous Pharmacokinetics or Lymphatic Exposure of Trastuzumab in Rats but Increases Plasma Clearance after Subcutaneous Administration [J]. 2015, - 12(- 3): - 809. | Irrelevant study |  |
| [18] | Changra H O, Diaz J F R. - Costs of intravenous vs. subcutaneous administration of trastuzumab in peruvian patients with HER2-positive breast cancer - An observational analysis of direct and indirect costs [J]. 2022, - 37(- 3): - 154. | The outcome indicators do not match |  |
| [19] | Cheal S M, Xu H, Guo H F, Patel M, Punzalan B, Fung E K, et al. Theranostic pretargeted radioimmunotherapy of internalizing solid tumor antigens in human tumor xenografts in mice: Curative treatment of HER2-positive breast carcinoma [J]. Theranostics, 2018, 8(18): 5106-5125. | Irrelevant study |  |
| [20] | Cheal S M, Xu H, Guo H-f, Patel M, Punzalan B, Fung E K, et al. - Theranostic pretargeted radioimmunotherapy of internalizing solid tumor antigens in human tumor xenografts in mice: Curative treatment of HER2-positive breast carcinoma [J]. 2018, - 8(- 18): - 5125. | Irrelevant study |  |
| [21] | Chen S C, Quartino A, Polhamus D, Riggs M, French J, Wang X, et al. Population pharmacokinetics and exposure-response of trastuzumab emtansine in advanced breast cancer previously treated with ≥2 HER2-targeted regimens [J]. British journal of clinical pharmacology, 2017, 83(12): 2767‐2777. | Irrelevant study |  |
| [22] | Cheng Y C, Valero V, Davis M L, Gonzalez-Angulo A M, Theriault R L, Murray J L, et al. - Addition of GM-CSF to trastuzumab stabilises disease in trastuzumab-resistant HER2+ metastatic breast cancer patients [J]. 2010, - 103(- 9): - 1334. | Irrelevant study |  |
| [23] | Cheng Y C, Valero V, Davis M L, Hortobagyi G N, Ueno N T. - Addition of Granulocyte-Macrophage Colony-Stimulating Factor (GM-CSF) to Trastuzumab Stabilizes Disease in Patients with Trastuzumab-Resistant, HER2+Metastatic Breast Cancer [J]. 2009, - 69(- 24): - 800S. | Irrelevant study |  |
| [24] | Chiradoni Thungappa S, Maksud T, Raut N, Nagarkar R, Batra U, Kumar S, et al. Comparison of the Efficacy, Safety, Pharmacokinetic and Immunogenicity of UJVIRA (ZRC-3256, Trastuzumab Emtansine) With the Kadcyla (Trastuzumab Emtansine) in the Treatment of HER2-Positive Metastatic Breast Cancer: a Randomized, Open-Label, Multicenter Study in India [J]. Clinical breast cancer, 2022, 22(4): 300‐307. | The research design is inconsistent |  |
| [25] | Cicin İ, Oukkal M, Mahfouf H, Mezlini A, Larbaoui B, Ahmed S B, et al. An Open-Label, Multinational, Multicenter, Phase IIIb Study with Subcutaneous Administration of Trastuzumab in Patients with HER2-Positive Early Breast Cancer to Evaluate Patient Satisfaction [J]. Meme Sagligi Dergisi / Journal of Breast Health, 2022, 18(1): 63-73. | The outcome indicators do not match |  |
| [26] | Cicin I, Oukkal M, Mahfouf H, Mezlini A, Larbaoui B, Slim B A, et al. An open-label, Multinational, Phase IIIb Study to Evaluate Patient and Satisfaction, Safety and Efficacy of Subcutaneous Administration of Trastuzumab in Patients with HER2-Positive Early Breast Cancer (ML28851) in Adjuvant/Neo-Adjuvant Setting [J]. European Journal of Cancer, 2018, 92: S102-S103. | The outcome indicators do not match |  |
| [27] | Ciruelos E, Gonzalez E, Lluch A, Garrigos L, Quiroga V, Anton A, et al. - Phase III clinical trial to evaluate patient's preference for subcutaneous (SC) versus intravenous (IV) trastuzumab administration in patients with HER2 positive, advanced breast cancer (ABC) under IV trastuzumab treatment for at least 4 months and without disease progression. ChangHER-SC study (GEICAM/2012-07) [J]. 2016, - 76. | The type of the research does not match. |  |
| [28] | Ciruelos E M, Montaño A, Rodríguez C A, González-Flores E, Lluch A, Garrigós L, et al. Phase III study to evaluate patient's preference of subcutaneous versus intravenous trastuzumab in HER2-positive metastatic breast cancer patients: results from the ChangHER study (GEICAM/2012-07) [J]. European journal of cancer care, 2020, 29(4): e13253. | The type of the research does not match. |  |
| [29] | Ciruelos E M, Montano A, Rodriguez C A, Gonzalez-Flores E, Lluch A, Garrigos L, et al. - Phase III trial to evaluate patient's preference for subcutaneous versus intravenous trastuzumab administration in patients with HER2 positive advanced breast cancer (ABC) under IV trastuzumab (IV-t) treatment for at least 4 months. ChangHER-SC study (GEICAM/2012-07) [J]. 2017, - 77. | The type of the research does not match. |  |
| [30] | Costantini D L, McLarty K, Lee H, Done S J, Vallis K A, Reilly R M. Antitumor effects and normal-tissue toxicity of 111In-nuclear localization sequence-trastuzumab in athymic mice bearing HER-positive human breast cancer xenografts [J]. Journal of nuclear medicine : official publication, Society of Nuclear Medicine, 2010, 51(7): 1084-1091. | The research object is not in line. |  |
| [31] | Cunha A. - Hospital Capacity Tool to Assess the Potential Impact of Using a Fixed Dose Combination of Pertuzumab/Trastuzumab for Subcutaneous Injection Versus Intravenous Pertuzumab/ Trastuzumab in Her2+Breast Cancer [J]. 2023, - 26(- 12): - S296. | The research design is inconsistent |  |
| [32] | Cunha A. HSD11 Hospital Capacity Tool to Assess the Potential Impact of Using a Fixed Dose Combination of Pertuzumab/Trastuzumab for Subcutaneous Injection Versus Intravenous Pertuzumab/Trastuzumab in HER2+ Breast Cancer [J]. Value in Health, 2023, 26(12): S296. | The research design is inconsistent |  |
| [33] | Dang C T, Tolaney S M, Riaz F, Tan A R, Tkaczuk K H R, Yu A F, et al. Preliminary analysis of an expanded access study of the fixed-dose combination of pertuzumab (P) and trastuzumab (H) for subcutaneous injection (PH FDC SC) for athome administration (admin) in patients (pts) with HER2-positive (HER2+) breast cancer (BC) during the COVID-19 pandemic [J]. Journal of Clinical Oncology, 2022, 40(16). | The research design is inconsistent |  |
| [34] | de Azambuja E, Holmes A P, Piccart-Gebhart M, Holmes E, Di Cosimo S, Swaby R F, et al. Lapatinib with trastuzumab for HER2-positive early breast cancer (NeoALTTO): survival outcomes of a randomised, open-label, multicentre, phase 3 trial and their association with pathological complete response [J]. The Lancet Oncology, 2014, 15(10): 1137‐1146. | The research design is inconsistent |  |
| [35] | De Cock E, Knoop A, Jakobsen E H, Ribecco A S, Hauser N, Kritikou P, et al. - Manual injection of subcutaneous trastuzumab vs intravenous infusion for HER2-positive early breast cancer: a time-and-motion study [J]. 2013, - 49: - S433. | The research design is inconsistent |  |
| [36] | De Cock E, Pivot X, Hauser N, Verma S, Kritikou P, Millar D, et al. A time and motion study of subcutaneous versus intravenous trastuzumab in patients with HER2-positive early breast cancer [J]. Cancer medicine, 2016, 5(3): 389-397. | The research design is inconsistent |  |
| [37] | de Cock E, Tao S, Alexa U, Pivot X, Knoop A. - Time savings with trastuzumab subcutaneous (SC) injection vs. trastuzumab intravenous (IV) infusion: First results from a Time-and-Motion study (T&M) [J]. 2012, - 72. | The research design is inconsistent |  |
| [38] | De Sanctis R, Giordano L, D'Antonio F, Agostinetto E, Marinello A, Guiducci D, et al. Clinical predictors of cardiac toxicity in HER2-positive early breast cancer patients treated with adjuvant s.c. versus i.v. trastuzumab [J]. Breast (Edinburgh, Scotland), 2021, 57: 80-85. | The research design is inconsistent |  |
| [39] | Dent R, Clemons M. Trastuzumab after primary treatment for early stage HER2-positive breast cancer reduces recurrence [J]. Cancer treatment reviews, 2006, 32(2): 144‐148. | The research design is inconsistent |  |
| [40] | Dent S, Ammendolea C, Christofides A, Edwards S, Incekol D, Pourmirza B, et al. - A multidisciplinary perspective on the subcutaneous administration of trastuzumab in HER2-positive breast cancer [J]. 2019, - 26(- 1): - E80. | The research design is inconsistent |  |
| [41] | Denys H, Martinez-Mena C L, Martens M T, D'Hondt R G, Graas M-P L, Evron E, et al. - Safety and tolerability of subcutaneous trastuzumab at home administration, results of the phase IIIb open-label BELIS study in HER2-positive early breast cancer [J]. 2020, - 181(- 1): - 105. | The research design is inconsistent |  |
| [42] | Duco M R, Murdock J L, Reeves D J. Trastuzumab/Hyaluronidase-oysk: A New Option for Patients With HER2-Positive Breast Cancer [J]. The Annals of pharmacotherapy, 2020, 54(3): 254-261. | The research design is inconsistent |  |
| [43] | DuMond B, Patel V, Gross A, Fung A, Weber S. - Fixed-dose combination of pertuzumab and trastuzumab for subcutaneous injection in patients with HER2-positive breast cancer: A multidisciplinary approach [J]. 2021, - 27(- 5): - 1221. | The measures do not match the comparison. |  |
| [44] | E. R, P. K W, H. N I, S. H, C. F Y, T. B-C, et al. - Treating human epidermal growth factor receptor 2 (HER2)-positive cancer, e.g. early breast cancer, by administering loading dose of fixed dose combination (FDC) of pertuzumab, trastuzumab, and recombinant human hyaluronidase (rHuPH20) [J]. | Irrelevant study |  |
| [45] | Earl H M, Hiller L, Vallier A L, Loi S, McAdam K, Hughes-Davies L, et al. 6 versus 12 months of adjuvant trastuzumab for HER2-positive early breast cancer (PERSEPHONE): 4-year disease-free survival results of a randomised phase 3 non-inferiority trial [J]. Lancet (London, England), 2019, 393(10191): 2599‐2612. | The measures do not match the comparison |  |
| [46] | Elsamany S, Elsisi G H, Hassanin F, Jafal M. Budget impact analysis of subcutaneous trastuzumab compared to intravenous trastuzumab in Saudi HER2-positive breast cancer patients [J]. Expert review of pharmacoeconomics & outcomes research, 2021, 21(3): 511-518. | The measures do not match the comparison |  |
| [47] | Emens L A, Esteva F J, Beresford M, Saura C, De Laurentiis M, Kim S B, et al. Trastuzumab emtansine plus atezolizumab versus trastuzumab emtansine plus placebo in previously treated, HER2-positive advanced breast cancer (KATE2): a phase 2, multicentre, randomised, double-blind trial [J]. The Lancet Oncology, 2020, 21(10): 1283‐1295. | The measures do not match the comparison. |  |
| [48] | Epstein R S. Payer perspectives on intravenous versus subcutaneous administration of drugs [J]. ClinicoEconomics and Outcomes Research, 2021, 13: 801-807. | Irrelevant study |  |
| [49] | Fallowfield L, Jenkins V, Kilkerr J, Langridge C, Monson K, Fitzpatrick N, et al. - Reasons for patients' preferences for subcutaneous or intravenous trastuzumab in the PrefHer study [J]. 2013, - 49: - S385. | The type of the research does not match. |  |
| [50] | Fallowfield L, Osborne S, Langridge C, Monson K, Kilkerr J, Jenkins V. - Implications of subcutaneous or intravenous delivery of trastuzumab; further insight from patient interviews in the PrefHer study [J]. 2015, - 24(- 2): - 170. | The type of the research does not match. |  |
| [51] | Feng Y, Meshaw R, Zhao X-G, Jannetti S, Vaidyanathan G, Zalutsky M R. - Effective Treatment of Human Breast Carcinoma Xenografts with Single-Dose <SUP>211</SUP>At-Labeled Anti-HER2 Single-Domain Antibody Fragment [J]. 2023, - 64(- 1): - 130. | Irrelevant study |  |
| [52] | Figallo M, Delgado M F, Gonzalez M, Arenas A. Cost minimization analysis of treatments for metastatic HER2-positive breast cancer: Fixed-Dose combination of pertuzumab and trastuzumab for subcutaneous injections [Z]. 2023.10.1101/2023.12.05.23299511 | The outcome indicators do not conform. |  |
| [53] | Figallo M, Delgado M F, Gonzalez M, Arenas A. Cost minimization analysis of treatments for metastatic HER2-positive breast cancer in Peru: Fixed-dose combination of pertuzumab and trastuzumab for subcutaneous injections [J]. PloS one, 2024, 19(11): e0295730. | The research design is inconsistent |  |
| [54] | Filippi M, Grimaldi L, Conte A, Totaro R, Valente M R, Malucchi S, et al. - Intravenous or subcutaneous natalizumab in patients with relapsing-remitting multiple sclerosis: investigation on efficiency and savings-the EASIER study [J]. 2024, - 271(- 1): - 354. | Irrelevant study |  |
| [55] | Franken M, Kanters T, Coenen J, de Jong P, Jager A, Groot C U D. Hospital-based or home-based administration of oncology drugs? A micro-costing study comparing healthcare and societal costs of hospital-based and home-based subcutaneous administration of trastuzumab [J]. Breast (Edinburgh, Scotland), 2020, 52: 71-77. | The research design is inconsistent |  |
| [56] | Gao J J, Osgood C L, Gong Y, Zhang H, Bloomquist E W, Jiang X, et al. FDA Approval Summary: Pertuzumab, Trastuzumab, and Hyaluronidase-zzxf Injection for Subcutaneous Use in Patients with HER2-positive Breast Cancer [J]. Clinical cancer research : an official journal of the American Association for Cancer Research, 2021, 27(8): 2126-2129. | The type of the research does not match. |  |
| [57] | Gavin P G, Kim P S, Lipchick C, Langley E, Feng H, Meyer G R, et al. An exploratory correlative biomarker analysis of NSABP FB-7, a phase II randomized trial evaluating neoadjuvant therapy with weekly paclitaxel (P) plus neratinib (N) or trastuzumab (T) or neratinib and trastuzumab (N+T) followed by doxorubicin and cyclophosphamide (AC) with postoperative T in women with locally advanced HER2-positive breast cancer [J]. Cancer Research, 2017, 77(4). | Irrelevant study |  |
| [58] | Gianni L, Eiermann W, Semiglazov V, Manikhas A, Lluch A, Tjulandin S, et al. Neoadjuvant chemotherapy with trastuzumab followed by adjuvant trastuzumab versus neoadjuvant chemotherapy alone, in patients with HER2-positive locally advanced breast cancer (the NOAH trial): a randomised controlled superiority trial with a parallel HER2-negative cohort [J]. Lancet (London, England), 2010, 375(9712): 377‐384. | Irrelevant study |  |
| [59] | Gligorov J, Curigliano G, Mueller V, Knoop A, Jenkins V, Osborne S, et al. - Assessment of adverse events in patients switching between trastuzumab administration routes (subcutaneous to intravenous and intravenous to subcutaneous) in the PrefHer study [J]. 2014, - 50: - S46. | The type of the research does not match. |  |
| [60] | Gligorov J, Pivot X, Ataseven B, De Laurentiis M, Llombart A, Jung K H, et al. - 5-year follow-up of the safety, tolerability and efficacy of subcutaneous trastuzumab for the adjuvant treatment of HER2-positive early breast cancer. Results from the SafeHER Phase III trial [J]. 2021, - 56: - S22. | The type of the research does not match. |  |
| [61] | Gregori J G, Miranda E L, Escriva-de-Romani S, Rodriguez B J, Novoa S A, Morales L F, et al. - Time and motion study of a subcutaneous fixed-dose combination of pertuzumab and trastuzumab for the treatment of patients with HER2-positive early breast cancer (PHaTiMa) [J]. 2022, - 82(- 4). | The research design is inconsistent |  |
| [62] | Hainfeld J F, O'Connor M J, Dilmanian F A, Slatkin D N, Adams D J, Smilowitz H M. - Micro-CT enables microlocalisation and quantification of Her2-targeted gold nanoparticles within tumour regions [J]. 2011, - 84(- 1002): - 533. | Irrelevant study |  |
| [63] | Hamizi S, Freyer G, Bakrin N, Henin E, Mohtaram A, Le Saux O, et al. - Subcutaneous trastuzumab: development of a new formulation for treatment of HER2-positive early breast cancer [J]. 2013, - 6: - 94. | The type of the research does not match. |  |
| [64] | Han H S, Costa R, Armaghani A, Soyano A, Loftus L, Soliman H, et al. Phase II neoadjuvant trial of Interferon-gamma plus weekly paclitaxel, trastuzumab and pertuzumab in patients with HER-2 positive breast cancer [J]. Cancer Research, 2022, 82(4 SUPPL). | Irrelevant study |  |
| [65] | Han H S, Khong H, Costa R, Loftus L, Goodridge D, Henry T, et al. A phase I study of interferon-gamma (Y)plus weekly paclitaxel, trastuzumab and pertuzumab in patients with HER-2 positive breast cancer [J]. Cancer Research, 2019, 79(4). | Irrelevant study |  |
| [66] | Harding S, Borley A. - Switching to a Fixed-dose Combined Pertuzumab and Trastuzumab With Recombinant Human Hyaluronidase Subcutaneous Injection to Treat Human Epidermal Growth Factor Receptor 2-positive Breast Cancer in Real-world UK Clinical Practice [J]. 2025, - 37. | The type of the research does not match. |  |
| [67] | Hedayati E, Fracheboud L, Srikant V, Greber D, Wallberg S, Stragliotto C L. - Economic benefits of subcutaneous trastuzumab administration: A single institutional study from Karolinska University Hospital in Sweden [J]. 2019, - 14(- 2). | The outcome indicators do not conform. |  |
| [68] | Heo Y A, Syed Y Y. Subcutaneous Trastuzumab: A Review in HER2-Positive Breast Cancer [J]. Targeted oncology, 2019, 14(6): 749-758. | The type of the research does not match. |  |
| [69] | Hurvitz S A, Andre F, Jiang Z, Shao Z, Mano M S, Neciosup S P, et al. Combination of everolimus with trastuzumab plus paclitaxel as first-line treatment for patients with HER2-positive advanced breast cancer (BOLERO-1): a phase 3, randomised, double-blind, multicentre trial [J]. The Lancet Oncology, 2015, 16(7): 816‐829. | Irrelevant study |  |
| [70] | Hurvitz S A, Hegg R, Chung W P, Im S A, Jacot W, Ganju V, et al. Trastuzumab deruxtecan versus trastuzumab emtansine in patients with HER2-positive metastatic breast cancer: updated results from DESTINY-Breast03, a randomised, open-label, phase 3 trial [J]. Lancet (London, England), 2023, 401(10371): 105‐117. | The research design is inconsistent |  |
| [71] | Im S A, Tan A R, Mattar A, Colomer R, Stroyakovskii D, Nowecki Z, et al. 46P Fixed-dose combination of pertuzumab and trastuzumab for subcutaneous injection (PH FDC SC) plus chemotherapy in HER2-positive early breast cancer (EBC): Safety results from the adjuvant phase of the randomised, open-label, multicentre phase III (neo)adjuvant FeDeriCa study [J]. Annals of Oncology, 2021, 32: S40-S41. | The research design is inconsistent |  |
| [72] | Inotai A, Ágh T, Karpenko A W, Zemplényi A, Kaló Z. Behind the subcutaneous trastuzumab hype: evaluation of benefits and their transferability to Central Eastern European countries [J]. Expert review of pharmacoeconomics & outcomes research, 2019, 19(2): 105-113. | The research design is inconsistent |  |
| [73] | Jackisch C, Hegg R, Stroyakovskiy D, Ahn J S, Melichar B, Chen S C, et al. - Phase III HannaH study of subcutaneous or intravenous trastuzumab for HER2-positive early breast cancer: Exploratory subgroup analyses of pathological complete response and 3-year event-free survival according to body weight and anti-drug antibody status [J]. 2015, - 51: - S314. | The research design is inconsistent |  |
| [74] | Jackisch C, Hegg R, Stroyakovskiy D, Ahn J S, Melichar B, Chen S-C, et al. - Total pathologic complete response (tpCR) and event-free survival (EFS) with subcutaneous (SC) or intravenous (IV) trastuzumab in HER2-positive early breast cancer (EBC) [J]. 2015, - 33(- 15). | No control measures |  |
| [75] | Jackisch C, Hegg R, Stroyakovskiy D, Ahn J-S, Melichar B, Chen S-C, et al. - HannaH phase III randomised study: Association of total pathological complete response with event-free survival in HER2-positive early breast cancer treated with neoadjuvant-adjuvant trastuzumab after 2 years of treatment-free follow-up [J]. 2016, - 62: - 75. | The research design is inconsistent |  |
| [76] | Jackisch C, Im S A, Mattar A, Bosch R C, Stroyakovskiy D, Nowecki Z, et al. Fixed-dose combination of pertuzumab and trastuzumab for subcutaneous injection (PH FDC SC) plus chemotherapy in HER2-positive early breast cancer (EBC): Long-term efficacy and safety analysis of the randomised, open-label, multicentre phase III (neo)adjuvant FeDeriCa study [J]. ESMO Open, 2024, 9. | The comparison with the measures does not conform. |  |
| [77] | Jackisch C, Kim S B, Semiglazov V, Melichar B, Pivot X, Hillenbach C, et al. Subcutaneous versus intravenous formulation of trastuzumab for HER2-positive early breast cancer: updated results from the phase III HannaH study [J]. Annals of oncology : official journal of the European Society for Medical Oncology, 2015, 26(2): 320‐325. | The type of the research does not match. |  |
| [78] | Jackisch C, Manevy F, Frank S, Roberts N, Shafrin J. White Paper on the Value of Time Savings for Patients and Healthcare Providers of Breast Cancer Therapy: The Fixed-Dose Combination of Pertuzumab and Trastuzumab for Subcutaneous Injection as an Example [J]. Advances in therapy, 2022, 39(2): 833-844. | The type of the research does not match. |  |
| [79] | Jackisch C, Stroyakovskiy D, Muehlbauer S, Heinzmann D, Kopp M V, Ahn J S, et al. - Subcutaneous Administration of Trastuzumab in Patients with HER2-positive Early Breast Cancer: Results From the Phase III Randomised, Open-label, Multi-centre Neoadjuvant-adjuvant HannaH Study [J]. 2012, - 48: - S37. | No control measures |  |
| [80] | Jackisch C, Stroyakovskiy D, Pivot X, Ahn J S, Melichar B, Chen S-C, et al. - Subcutaneous vs Intravenous Trastuzumab for Patients With ERBB2-Positive Early Breast Cancer Final Analysis of the HannaH Phase 3 Randomized Clinical Trial [J]. 2019, - 5(- 5). | The type of the research does not match. |  |
| [81] | Jackisch C, Stroyakovskiy D, Pivot X, Ahn J-S, Melichar B, Chen S-C, et al. - Efficacy and safety of subcutaneous or intravenous trastuzumab in patients with HER2-positive early breast cancer after 5 years' treatment-free follow-up: Final analysis from the phase III, open-label, randomized HannaH study [J]. 2018, - 78(- 4). | The type of the research does not match. |  |
| [82] | Johnson M L, Braiteh F, Grilley-Olson J E, Chou J, Davda J, Forgie A, et al. - Assessment of Subcutaneous vs Intravenous Administration of Anti-PD-1 Antibody PF-06801591 in Patients With Advanced Solid Tumors A Phase 1 Dose-Escalation Trial [J]. 2019, - 5(- 7): - 1007. | Irrelevant study |  |
| [83] | Kaidarova D, Zhavrid E, Shatkovskaya O, Prokharau A, Akhmed N, Sembayev D, et al. Assessment of safety and tolerability of subcutaneous trastuzumab in patients with HER2-positive early breast cancer: Results of an open-label, randomized, multicenter, phase IIIB ESCAPE trial [J]. Cancer treatment and research communications, 2024, 40: 100817. | No control measures |  |
| [84] | Kang L, Raitman I, Rotondo S, Gleason J, He S, Somanchi S, et al. - Human Placental CD34<SUP>+</SUP>-Derived Natural Killer Cells with High Affinity and Cleavage Resistant CD16 (CYNK-101) for ADCC Mediated Cancer Immunotherapy [J]. 2020, - 136. | Irrelevant study |  |
| [85] | Karmali S, Hughes N, Kinneally A, Kroes J, Cook J, Killian M, et al. - A regional audit of 6-hour monitoring for administration related reactions during the first administration of subcutaneous trastuzumab [J]. 2019, - 79(- 4). | Irrelevant study |  |
| [86] | Kaufman B, Mackey J R, Clemens M R, Bapsy P P, Vaid A, Wardley A, et al. Trastuzumab plus anastrozole versus anastrozole alone for the treatment of postmenopausal women with human epidermal growth factor receptor 2-positive, hormone receptor-positive metastatic breast cancer: results from the randomized phase III TAnDEM study [J]. Journal of clinical oncology, 2009, 27(33): 5529‐5537. | Irrelevant study |  |
| [87] | Khine M, Mon S, Sein N, Aye T T, Htay S S, Kyaw N H, et al. Molecular targeted therapy for Her2 positive breast cancer in private sector: Yangon experience [J]. Annals of Oncology, 2016, 27: ix23. | Irrelevant study |  |
| [88] | Kim H, Alten R, Cummings F, Danese S, D'Haens G, Emery P, et al. - Innovative approaches to biologic development on the trail of CT-P13: biosimilars, value-added medicines, and biobetters [J]. 2021, - 13(- 1). | Irrelevant study |  |
| [89] | Kirschbrown W P, Wynne C, Kågedal M, Wada R, Li H, Wang B, et al. Development of a Subcutaneous Fixed-Dose Combination of Pertuzumab and Trastuzumab: Results From the Phase Ib Dose-Finding Study [J]. Journal of clinical pharmacology, 2019, 59(5): 702-716. | The research design is inconsistent |  |
| [90] | Kokkali S, Ternant D, Kemmel V, Levêque D, Wendling F, Barthelemy P, et al. Intravenous and subcutaneous administration of trastuzumab in a patient on peritoneal dialysis [J]. British Journal of Clinical Pharmacology, 2021, 87(8): 3372-3374. | Irrelevant study |  |
| [91] | Kolberg H C, Jackisch C, Hurvitz S A, Winstone J, Barham H, Hanes V, et al. Is weight-based IV dosing of trastuzumab preferable to SC fixed-dose in some patients? A systematic scoping review [J]. Breast (Edinburgh, Scotland), 2021, 57: 95-103. | The type of the research does not match. |  |
| [92] | Kuemmel S, Harper-Wynne C, Park Y H, Franke F, de Laurentiis M, Schumacher-Wulf E, et al. heredERA Breast Cancer: a phase III, randomized, open-label study evaluating the efficacy and safety of giredestrant plus the fixed-dose combination of pertuzumab and trastuzumab for subcutaneous injection in patients with previously untreated HER2-positive, estrogen receptor-positive locally advanced or metastatic breast cancer [J]. BMC Cancer, 2024, 24(1). | Irrelevant study |  |
| [93] | Kuemmel S, Tondini C A, Abraham J, Nowecki Z, Itrych B, Hitre E, et al. - Subcutaneous trastuzumab and hyaluronidase-oysk with intravenous pertuzumab and docetaxel in HER2-positive advanced breast cancer: Final analysis of the phase IIIb, multicenter, open-label, single-arm MetaPHER study [J]. 2020, - 80(- 4). | The research design is inconsistent |  |
| [94] | Kuemmel S, Tondini C A, Abraham J, Nowecki Z, Itrych B, Hitre E, et al. - Subcutaneous trastuzumab with pertuzumab and docetaxel in HER2-positive metastatic breast cancer: Final analysis of MetaPHER, a phase IIIb single-arm safety study [J]. 2021, - 187(- 2): - 476. | The research design is inconsistent |  |
| [95] | Kuemmel S, Tondini C A, Abraham J, Nowecki Z, Itrych B, Hitre E, et al. Subcutaneous trastuzumab with pertuzumab and docetaxel in HER2-positive metastatic breast cancer: Final analysis of MetaPHER, a phase IIIb single-arm safety study [J]. Breast cancer research and treatment, 2021, 187(2): 467-476. | The research design is inconsistent |  |
| [96] | Kuemmel S, Tondini C A, Abraham J, Nowecki Z I, Itrych B, Hitre E, et al. - Subcutaneous trastuzumab (H SC) with intravenous pertuzumab (P IV) and docetaxel (D IV) in HER2-positive advanced breast cancer (BC): MetaPHER second interim analysis [J]. 2018, - 29: - 103. | The research design is inconsistent |  |
| [97] | Laird-Fick H S, Tokala H, Kandola S, Kehdi M, Pelosi A, Wang L, et al. Early morphological changes in cardiac mitochondria after subcutaneous administration of trastuzumab in rabbits: possible prevention with oral selenium supplementation [J]. Cardiovascular Pathology, 2020, 44. | Irrelevant study |  |
| [98] | Landeiro L C G, Martins T d C, Grigolon R B, Monteiro I, Balardin J B, Padilha E, et al. - The burden of systemic therapy administration route in treating HER2-positive breast cancer (for patients, healthcare professionals, and healthcare system): a systematic literature review [J]. 2024, - 15. | The type of the research does not match. |  |
| [99] | Launay-Vacher V. - An appraisal of subcutaneous trastuzumab: a new formulation meeting clinical needs [J]. 2013, - 72(- 6): - 1367. |  |  |
| [100] | Lazaro Cebas A, Cortijo Cascajares S, Pablos Bravo S, Del Puy Goyache Goñi M, Gonzalez Monterrubio G, Perez Cardenas M D, et al. Subcutaneous versus intravenous administration of trastuzumab: preference of HER2+ breast cancer patients and financial impact of its use [J]. Journal of BUON : official journal of the Balkan Union of Oncology, 2017, 22(2): 334-339. | The outcome indicators do not conform |  |
| [101] | Lee A L Z, Ng V W L, Gao S, Hedrick J L, Yang Y Y. - Injectable Hydrogels from Triblock Copolymers of Vitamin E- Functionalized Polycarbonate and Poly( ethylene glycol) for Subcutaneous Delivery of Antibodies for Cancer Therapy [J]. 2014, - 24(- 11): - 1550. | Irrelevant study |  |
| [102] | Lee V W Y, Cheng F W T. Cost-minimisation analysis of intravenous versus subcutaneous trastuzumab regimen for breast cancer management in Hong Kong [J]. Hong Kong medical journal = Xianggang yi xue za zhi, 2023, 29(1): 16-21. | The outcome indicators do not conform |  |
| [103] | Leyland-Jones B, Arnold A, Gelmon K, Verma S, Ayoub J P, Seidman A, et al. - Pharmacologic insights into the future of trastuzumab [J]. 2001, - 12: - 47. | Irrelevant study |  |
| [104] | Lieutenant V, Toulza E, Pommier M, Lortal-Canguilhem B. - Is Herceptin® (trastuzumab) by subcutaneous a mini revolution? Pharmaco-economic study [J]. 2015, - 102(- 3): - 276. | The type of the research does not match. |  |
| [105] | Lin H W, Lin C Y, Yeh T P, Lin T C, Yeh W C, Yang L C, et al. Quality of care in the course of subcutaneous versus intravenous trastuzumab administration in patients with breast cancer: an integrated time-motion study with mixed-methods research [J]. BMJ open, 2023, 13(3): e059288. | The research design is inconsistent |  |
| [106] | Liu S N, Lu T, Jin J Y, Li C, Girish S, Melnikov F, et al. Impact of Dose Delays and Alternative Dosing Regimens on Pertuzumab Pharmacokinetics [J]. Journal of clinical pharmacology, 2021, 61(8): 1096-1105. | Irrelevant study |  |
| [107] | Longo R, Thiebaut V, Legros P-O, Campitiello M, Plastino F, Goetz C, et al. - Is the TCH-P regimen active in early or locally advanced HER2-positive breast cancer? Results of a retrospective study [J]. 2022, - 61(- 11): - 1399. | The research design is inconsistent |  |
| [108] | Luo Y, Li W, Jiang Z, Zhang Q, Wang L, Mao Y, et al. Pharmacokinetics of pertuzumab administered concurrently with trastuzumab in Chinese patients with HER2-positive early breast cancer [J]. Anti-cancer drugs, 2019, 30(8): 866‐872. | The research design is inconsistent |  |
| [109] | Manevy F, Filkauskas G, Levy P, Fredriksson J, Sussell J. Potential non-drug cost differences associated with the use of the fixed-dose combination of pertuzumab and trastuzumab for subcutaneous injection (PH FDC SC) in the treatment of HER2-positive early breast cancer patients in Western Europe and the United States [J]. Journal of Clinical Oncology, 2021, 39(15 SUPPL). | The research design is inconsistent |  |
| [110] | Mani A, Roda J, Young D, Caligiuri M A, Fleming G F, Kaufman P, et al. - A phase II trial of trastuzumab in combination with low-dose interleukin-2 (IL-2) in patients (PTS) with metastatic breast cancer (MBC) who have previously failed trastuzumab [J]. 2009, - 117(- 1): - 89. | The research design is inconsistent |  |
| [111] | Manuel Perez-Garcia J, Gebhart G, Ruiz Borrego M, Stradella A, Bermejo B, Schmid P, et al. - Chemotherapy de-escalation using an <SUP>18</SUP>F-FDG-PET-based pathological response-adapted strategy in patients with HER2-positive early breast cancer (PHERGain): a multicentre, randomised, open-label, non-comparative, phase 2 trial [J]. 2021, - 22(- 6): - 871. | The research design is inconsistent |  |
| [112] | Marty C B, Blein C, Borg M C, Alfonsi R, Priou V, Tournamille J F, et al. - A multi-center evaluation of clinical pathways cost and time using real-life data in 411 breast cancer patients treated with intravenous versus subcutaneous Trastuzumab [J]. 2018, - 92: - S107. | The outcome indicators do not conform. |  |
| [113] | McBride A, MacDonald K, Fuentes-Alburo A, Abraham I. Cost-efficiency and expanded access modeling of conversion to biosimilar trastuzumab-dkst with or without pertuzumab in metastatic breast cancer [J]. J Med Econ, 2021, 24(1): 743-756. | The outcome indicators do not conform. |  |
| [114] | McCloskey C, Ortega M T, Nair S, Garcia M J, Manevy F. - A Systematic Review of Time and Resource Use Costs of Subcutaneous Versus Intravenous Administration of Oncology Biologics in a Hospital Setting [J]. 2023, - 7(- 1): - 36. | The type of the research does not match. |  |
| [115] | Medina E A G, Caballero B B, Miguel K L, Gutiérrez Z A, Fernández B M, Tul L E A, et al. Neoadjuvant Trastuzumab and Pertuzumab in Combination with Standard Chemotherapy for HER2-Positive Early Breast Cancer: Real-World Practice in Cuba [J]. Cancer treatment and research communications, 2023, 34: 100670. | The research design is inconsistent |  |
| [116] | Meirelles I, Nogueira Bezerra G, Monteiro I, Martins T. EE665 Evaluation of Resource Use and Costs of Pertuzumab and Trastuzumab Formulations in HER2- Positive Breast Cancer [J]. Value in Health, 2023, 26(12): S181. | The research design is inconsistent |  |
| [117] | Melichar B, Študentová H, Kalábová H, Vitásková D. Role of subcutaneous formulation of trastuzumab in the treatment of patients with HER2-positive breast cancer [J]. Immunotherapy, 2014, 6(7): 811-819. | The research design is inconsistent |  |
| [118] | Milenic D E, Wong K J, Baidoo K E, Nayak T K, Regino C A S, Garmestani K, et al. - Targeting HER2 A report on the in vitro and in vivo pre-clinical data supporting trastuzumab as a radioimmunoconjugate for clinical trials [J]. 2010, - 2(- 5): - 564. | Irrelevant study |  |
| [119] | Monteiro I, Cordeiro F, Martins T, Medeiros C. Impact Associated With Pertuzumab and Trastuzumab SC and IV Formulations Utilization in Metastatic HER2-Positive Breast Cancer Patients Treated in Brazilian SUS [Z]. 2024: S68.10.1016/j.jval.2024.10.360 | Irrelevant study |  |
| [120] | Moulder S L, Arteaga C L. A phase I/II trial of trastuzumab and gefitinib in patients with metastatic breast cancer that overexpresses HER2/neu (ErbB-2) [J]. Clinical breast cancer, 2003, 4(2): 142‐145. | The research design is inconsistent |  |
| [121] | Munzone E, Fabi A, Buono G, Caputo R, Montagna E, Negri M, et al. - The PHASTER Study: Economic and Organizational Impact of Subcutaneous (SC) Pertuzumab and Trastuzumab Fixed-Dose Combination (PH FDC SC) for Treatment of HER2+Breast Cancer Patients [J]. 2023. | The research design is inconsistent |  |
| [122] | Mylonas C, Kourlaba G, Fountzilas G, Skroumpelos A, Maniadakis N. - Cost-Minimization Analysis of Trastuzumab Intravenous Versus Trastuzumab Subcutaneous for the Treatment of Patients with Her2+Early Breast Cancer and Metastatic Breast Cancer in Greece [J]. 2014, - 17(- 7): - A641. | The outcome indicators do not conform. |  |
| [123] | Nawaz S, Samanta K, Lord S, Diment V, McNamara S. - Cost Savings with Herceptin® (Trastuzumab) Sc Vs Iv Administration: A Time & Motion Study [J]. 2013, - 22: - S113. | The outcome indicators do not conform. |  |
| [124] | Nct. Pre Operative Trastuzumab in Operable Breast Cancer [J]. https://clinicaltrialsgov/show/NCT01785420, 2013. | Irrelevant study |  |
| [125] | Nestorovska A, Naumoska Z, Grozdanova A, Stoleski D, Ivanovska A, Risteski M, et al. - Subcutaneous Vs Intravenous Administration of Trastuzumab in Her2+Breast Cancer Patients: A Macedonian Cost-Minimization Analysis [J]. 2015, - 18(- 7): - A463. | The outcome indicators do not conform. |  |
| [126] | Nguyen T, Tran D. - Cost Minimization Analysis of Trastuzumab Subcutaneous Versus Intravenous in Treatment of Her2-Positive Early or Metastatic Breast Cancer in Vietnam [J]. 2020, - 23: - S131. | The outcome indicators do not conform. |  |
| [127] | North R T, Harvey V J, Cox L C, Ryan S N. - Medical resource utilization for administration of trastuzumab in a New Zealand oncology outpatient setting: a time and motion study [J]. 2015, - 7: - 30. | Irrelevant study |  |
| [128] | Ntekim A I, Ibraheem A, Sofoluwe A A, Kotila O, Babalola C, Karrison T, et al. - ARETTA: Assessing Response to Neoadjuvant Taxotere and Subcutaneous Trastuzumab in Nigerian Women With HER2-Positive Breast Cancer: A Study Protocol [J]. 2020, - 6: - 990. | The research design is inconsistent |  |
| [129] | O'Brien G L, O'Mahony C, Cooke K, Kinneally A, Sinnott S-J, Walshe V, et al. - Cost Minimization Analysis of Intravenous or Subcutaneous Trastuzumab Treatment in Patients With HER2-Positive Breast Cancer in Ireland [J]. 2019, - 19(- 3): - E451. | The outcome indicators do not conform. |  |
| [130] | Olofsson S, Norrlid H, Karlsson E, Wilking U, Tennvall G R. - Societal cost of subcutaneous and intravenous trastuzumab for HER2-positive breast cancer - An observational study prospectively recording resource utilization in a Swedish healthcare setting [J]. 2016, - 29: - 146. | The outcome indicators do not conform. |  |
| [131] | Orlando L, Schiavone P, Calvani N, Fedele P, Goldhirsch A, Cinieri S. Response of extensive breast cancer skin metastases to rechallenge with trastuzumab together with low-dose chemotherapy and insulin [J]. Tumori, 2016, 102(Suppl. 2). | Irrelevant study |  |
| [132] | O'Shaughnessy J, Sousa S, Cruz J, Fallowfield L, Auvinen P, Pulido C, et al. Preference for the fixed-dose combination of pertuzumab and trastuzumab for subcutaneous injection in patients with HER2-positive early breast cancer (PHranceSCa): a randomised, open-label phase II study [J]. European journal of cancer (Oxford, England : 1990), 2021, 152: 223‐232. | The research design is inconsistent |  |
| [133] | O'Shaughnessy J, Sousa S, Cruz J, Fallowfield L, Auvinen P, Pulido C, et al. - Original Research Preference for the fixed-dose combination of pertuzumab and trastuzumab for subcutaneous injection in patients with HER2-positive early breast cancer (PHranceSCa): A randomised, open-label phase II study [J]. 2021, - 152: - 232. | The research design is inconsistent |  |
| [134] | O'Shaughnessy J, Sousa S P, Jurado J C, Fallowfield L J, Auvinen P, Pulido C, et al. 97P Efficacy and safety of the fixed-dose combination of pertuzumab and trastuzumab for subcutaneous injection in patients with HER2-positive early breast cancer: Long-term data from the PHranceSCa study [J]. ESMO Open, 2023, 8(1). | The research design is inconsistent |  |
| [135] | Otoya I, Valdivieso N, Morante Z, Castaneda C, Neciosup S, Calderon M, et al. - Safety and Tolerability of Subcutaneous Trastuzumab as a Treatment in Patients with Early Her 2 Positive Breast Cancer : Experience of a Cancer Center in Peru [J]. 2024, - 84(- 9). | No control measures |  |
| [136] | Otoya I, Valdivieso N, Morante Z, Castañeda C, Neciosup S, Calderón M, et al. SAFETY AND TOLERABILITY OF SUBCUTANEOUS TRASTUZUMAB AS A TREATMENT IN PATIENTS WITH EARLY HER 2 POSITIVE BREAST CANCER : EXPERIENCE OF A CANCER CENTER IN PERU [J]. Cancer Research, 2024, 84(9). | No control measures |  |
| [137] | Otoya I, Valdiviezo N, Morante Z, Calle C, Ferreyra Y, Huarcaya-Chombo N, et al. - Subcutaneous Trastuzumab: An Observational Study of Safety and Tolerability in Patients With Early HER2-Positive Breast Cancer [J]. 2024, - 2024. | No control measures |  |
| [138] | Otoya I, Valdiviezo N, Roque K, Morante Z, Vidaurre T, Neciosup S P, et al. Subcutaneous versus intravenous administration of Trastuzumab: a minimization cost analysis with real world data from a reference cancer centre in Peru [J]. ecancermedicalscience, 2024, 18. | The outcome indicators do not conform. |  |
| [139] | Ouyang Y, Lee H Y, Leong F L, Tey H J, Shih V, Lim E H, et al. - Cost-minimization analysis comparing subcutaneous trastuzumab at home with intravenous trastuzumab for HER2-positive breast cancer in Singapore [J]. 2024, - 16. | The outcome indicators do not conform. |  |
| [140] | Padrón I M, García J G, Díaz R R, Lenza I C, Nicolás F G. Anti-drug antibodies anti-trastuzumab in the treatment of breast cancer [J]. Journal of oncology pharmacy practice : official publication of the International Society of Oncology Pharmacy Practitioners, 2021, 27(6): 1354-1356. | Irrelevant study |  |
| [141] | Pallaro S, Bigas M, Leobon S, Baffert K A, Peyramaure C, Dubest L, et al. [Administration of anti-HER2 and satisfaction of patients treated for breast cancer] [J]. Bulletin du cancer, 2024, 111(5): 441-451. | Irrelevant study |  |
| [142] | Papadmitriou K, Trinh X B, Altintas S, Van Dam P A, Huizing M T, Tjalma W A A. - The socio-economical impact of intravenous (IV) versus subcutaneous (SC) administration of trastuzumab: future prospectives [J]. 2015, - 7(- 3): - 80. | The outcome indicators do not conform. |  |
| [143] | Parra A, Hernandez C, Prieto-Pinto L. - Evaluation of the economic benefits, administration times, and patient preferences associated with the use of biotechnological drugs administered subcutaneously and intravenously in patients with cancer: a systematic review [J]. 2023, - 23(- 9): - 1026. | The research type does not match. |  |
| [144] | Pegram M D, Bondarenko I, Zorzetto M M C, Hingmire S, Iwase H, Krivorotko P V, et al. PF-05280014 (a trastuzumab biosimilar) plus paclitaxel compared with reference trastuzumab plus paclitaxel for HER2-positive metastatic breast cancer: a randomised, double-blind study [J]. British journal of cancer, 2019, 120(2): 172‐182. | Irrelevant study |  |
| [145] | Penichet M L, Dela Cruz J S, Shin S U, Morrison S L. - A recombinant IgG3-(IL-2) fusion protein for the treatment of human HER2/neu expressing tumors [J]. 2001, - 10(- 1): - 9. | Irrelevant study |  |
| [146] | Pérez-García J M, Cortés J, Ruiz-Borrego M, Colleoni M, Stradella A, Bermejo B, et al. 3-year invasive disease-free survival with chemotherapy de-escalation using an (18)F-FDG-PET-based, pathological complete response-adapted strategy in HER2-positive early breast cancer (PHERGain): a randomised, open-label, phase 2 trial [J]. Lancet (London, England), 2024, 403(10437): 1649-1659. | Irrelevant study |  |
| [147] | Pérez-García J M, Gebhart G, Ruiz Borrego M, Stradella A, Bermejo B, Schmid P, et al. Chemotherapy de-escalation using an (18)F-FDG-PET-based pathological response-adapted strategy in patients with HER2-positive early breast cancer (PHERGain): a multicentre, randomised, open-label, non-comparative, phase 2 trial [J]. The Lancet Oncology, 2021, 22(6): 858-871. | Irrelevant study |  |
| [148] | Piccart M, Pinto A. - Patients with Her2 Positive Breast Cancer: Delivery, Duration and Combination Therapies [J]. 2013, - 22: - S17. | Irrelevant study |  |
| [149] | Pimentel F F, Morgan G, Tiezzi D G, de Andrade J M. - Development of New Formulations of Biologics: Expectations, Immunogenicity, and Safety for Subcutaneous Trastuzumab [J]. 2018, - 32(- 5): - 325. | Irrelevant study |  |
| [150] | Pinguet F, Milano G. - Subcutaneous dual-therapies: Methods of absorption and clinical implications [J]. 2014, - 16(- 7-8): - 400. | Irrelevant study |  |
| [151] | Pinto A C, Ades F, de Azambuja E, Piccart-Gebhart M. Trastuzumab for patients with HER2 positive breast cancer: delivery, duration and combination therapies [J]. Breast (Edinburgh, Scotland), 2013, 22 Suppl 2: S152-155. | The research design is inconsistent |  |
| [152] | Parra A, Hernandez C, Prieto-Pinto L. - Evaluation of the economic benefits, administration times, and patient preferences associated with the use of biotechnological drugs administered subcutaneously and intravenously in patients with cancer: a systematic review [J]. 2023, - 23(- 9): - 1026. | The research type does not match. |  |
| [153] | O'Brien G L, O'Mahony C, Cooke K, Kinneally A, Sinnott S-J, Walshe V, et al. - Cost Minimization Analysis of Intravenous or Subcutaneous Trastuzumab Treatment in Patients With HER2-Positive Breast Cancer in Ireland [J]. 2019, - 19(- 3): - E451. | The outcome indicators do not conform. |  |
| [154] | Penichet M L, Dela Cruz J S, Shin S U, Morrison S L. - A recombinant IgG3-(IL-2) fusion protein for the treatment of human HER2/neu expressing tumors [J]. 2001, - 10(- 1): - 9. | Irrelevant study |  |
| [155] | Pivot X, Manikhas A, Żurawski B, Chmielowska E, Karaszewska B, Allerton R, et al. CEREBEL (EGF111438): a Phase III, Randomized, Open-Label Study of Lapatinib Plus Capecitabine Versus Trastuzumab Plus Capecitabine in Patients With Human Epidermal Growth Factor Receptor 2-Positive Metastatic Breast Cancer [J]. Journal of clinical oncology, 2015, 33(14): 1564‐1573. | The measures do not match the comparison. |  |
| [156] | Pivot X, Romieu G, Debled M, Pierga J Y, Kerbrat P, Bachelot T, et al. 6 months versus 12 months of adjuvant trastuzumab for patients with HER2-positive early breast cancer (PHARE): a randomised phase 3 trial [J]. The Lancet Oncology, 2013, 14(8): 741‐748. | The research design is inconsistent |  |
| [157] | Pivot X, Romieu G, Debled M, Pierga J Y, Kerbrat P, Bachelot T, et al. 6 months versus 12 months of adjuvant trastuzumab in early breast cancer (PHARE): final analysis of a multicentre, open-label, phase 3 randomised trial [J]. Lancet (London, England), 2019, 393(10191): 2591‐2598. | The research design is inconsistent |  |
| [158] | Pivot X, Spano J-P, Espie M, Jouannaud C, Pottier V, Moreau L, et al. - Long Terms Follow-Up of the Randomized MetaspHER Study Comparing Intravenous Versus Subcutaneous Trastuzumab in Patients' With HER2-Positive Metastatic Breast Cancer [J]. 2023, - 23(- 7): - e419. | The research design is inconsistent |  |
| [159] | Pivot X, Verma S, Fallowfield L, Mueller V, Lichinitser M, Sanchez Munoz A, et al. - Efficacy and safety of subcutaneous trastuzumab and intravenous trastuzumab as part of adjuvant therapy for HER2-positive early breast cancer: final analysis of the randomised, two-cohort PrefHer study [J]. 2016, - 27. | The research type does not match. |  |
| [160] | Quartino A, Li J, Li H, Wada D R, Visich J, Li C, et al. - Population pharmacokinetic (PK) analysis of trastuzumab confirms the appropriateness of a fixed (non-weight-based) subcutaneous (SC) formulation dose in patients with HER2-positive early breast cancer (EBC) [J]. 2014, - 50: - S44. | Irrelevant study |  |
| [161] | Quartino A L, Hillenbach C, Li J, Li H, Wada R D, Visich J, et al. Population pharmacokinetic and exposure-response analysis for trastuzumab administered using a subcutaneous "manual syringe" injection or intravenously in women with HER2-positive early breast cancer [J]. Cancer chemotherapy and pharmacology, 2016, 77(1): 77-88. | Irrelevant study |  |
| [162] | Quiroga D, Wesolowski R, Zelinskas S, Pinette A, Benner B, Schwarz E, et al. An Open-Label Study of Subcutaneous CpG Oligodeoxynucleotide (PF03512676) in Combination with Trastuzumab in Patients with Metastatic HER2+ Breast Cancer [J]. Cancer Control, 2024, 31. | Irrelevant study |  |
| [163] | Radecka B, Hudala-Klecha J, Sawka D, Sarga J, Noworolska B, Susczyk G, et al. - Home-based treatment with subcutaneous trastuzumab: safe and acceptable not only during a pandemic - final analysis of the RWD project 'FlexCare' [J]. 2024, - 20(- 2): - 123. | Irrelevant study |  |
| [164] | Reilly R M, Georgiou C J, Brown M K, Cai Z. - Radiation nanomedicines for cancer treatment: a scientific journey and view of the landscape [J]. 2024, - 9(- 1). | Irrelevant study |  |
| [165] | Reinisch M, Untch M, Mahlberg R, Reimer T, Hitschold T, Marmé F, et al. Subcutaneous injection of trastuzumab into the thigh versus abdominal wall in patients with HER2-positive early breast cancer: Pharmacokinetic, safety and patients' preference - Substudy of the randomised phase III GAIN-2 study [J]. Breast (Edinburgh, Scotland), 2022, 66: 110-117. | The research design is inconsistent |  |
| [166] | Roda J, Parihar R, Lamb T, Bekaii-Saab T, Carson W E. - A phase I trial of interleukin-12 with trastuzumab and paclitaxel for HER2-overexpressing malignancies [J]. 2005, - 28(- 6): - 644. | Irrelevant study |  |
| [167] | Rodriguez F C, Castillo-Fernandez O, Lim-Law M, Palacios C M, Montano L, Benitez S, et al. - Real-world data of subcutaneous trastuzumab and intravenous pertuzumab as neoadjuvant therapy for localized HER2+ breast cancer in Panama [J]. 2021, - 10(- 1). | Irrelevant study |  |
| [168] | Rojas L, Muniz S, Medina L, Pena J, Acevedo F, Pinto M P, et al. - Cost-minimization analysis of subcutaneous versus intravenous trastuzumab administration in Chilean patients with HER2-positive early breast cancer [J]. 2020, - 15(- 2). | The outcome indicators do not conform. |  |
| [169] | Rojas L, Muñiz S, Medina L, Peña J, Acevedo F, Pinto M P, et al. Cost-minimization analysis of subcutaneous versus intravenous trastuzumab administration in Chilean patients with HER2-positive early breast cancer [J]. PloS one, 2020, 15(2): e0227961. | The outcome indicators do not conform. |  |
| [170] | Rugo H S, Barve A, Waller C F, Hernandez-Bronchud M, Herson J, Yuan J, et al. Effect of a Proposed Trastuzumab Biosimilar Compared With Trastuzumab on Overall Response Rate in Patients With ERBB2 (HER2)-Positive Metastatic Breast Cancer: a Randomized Clinical Trial [J]. Jama, 2017, 317(1): 37‐47. | The research design is inconsistent |  |
| [171] | Sakaeda M, Kotani N, Yoneya T, Zheng Y, Habara Y. [Pharmacological properties and clinical development overview of pertuzumab (genetical recombination), trastuzumab (genetical recombination) and vorhyaluronidase alfa (genetical recombination) (PHESGO(®) combination for ‍subcutaneous injection MA, IN)] [J]. Nihon yakurigaku zasshi Folia pharmacologica Japonica, 2024, 159(4): 241-253. | Irrelevant study |  |
| [172] | Sakaeda M, Kotani N, Yoneya T, Zheng Y, Habara Y. Pharmacological properties and clinical development overview of pertuzumab (genetical recombination), trastuzumab (genetical recombination) and vorhyaluronidase alfa (genetical recombination) (PHESGO® combination for subcutaneous injection MA, IN) [J]. Folia Pharmacologica Japonica, 2024, 159(4): 241-253. | Irrelevant study |  |
| [173] | Sampath L, Kwon S, Ke S, Wang W, Schiff R, Mawad M E, et al. - Dual-labeled trastuzumab-based imaging agent for the detection of human epidermal growth factor receptor 2 overexpression in breast cancer [J]. 2007, - 48(- 9): - 1510. | Irrelevant study |  |
| [174] | Sanchez-Bayona R, del Barrio M G, Alegre E, Fernandez-Hidalgo O A, Eslava M S. - Trastuzumab and thyroid dysfunction: An association to be aware of [J]. 2022, - 18(- 4): - 1185. | Irrelevant study |  |
| [175] | Sánchez-Bayona R, Garcia Del Barrio M A, Alegre E, Fernandez-Hidalgo O A, Eslava M S. Trastuzumab and thyroid dysfunction: An association to be aware of [J]. Journal of cancer research and therapeutics, 2022, 18(4): 1183-1185. | Irrelevant study |  |
| [176] | Sanford M. - Subcutaneous trastuzumab: a review of its use in HER2-positive breast cancer [J]. 2014, - 9(- 1): - 94. | The type of the research does not match. |  |
| [177] | Saric N C, Lekic S, Lakicevic J, Todorovic V. - Impact of subcutaneous versus intravenous administration of pertuzumab and trastuzumab (PH) for the treatment of HER2-positive breast cancer in Montenegro [J]. 2022, - 175: - S72. | The research design is inconsistent |  |
| [178] | Saure Sarría V M, Arencibia A D, D'Afonseca V. Xeloda Oral, Trastuzumab, and Pertuzumab Combined Drug Therapy Reduced Cervical Lymphadenopathy and Dermal Involvement in Patient With Recurrent Breast Cancer: Case Report [J]. Journal of investigative medicine high impact case reports, 2020, 8: 2324709620942606. | The type of the research does not match. |  |
| [179] | Schmidt M, KÜmmel S, Ruf-Doerdelmann A, Distelrath A, Wacker J, Schmatloch S, et al. Neo-adjuvant and/or Adjuvant Subcutaneous Trastuzumab (Herceptin(®)) in Patients With Early HER2-positive Breast Cancer: Real World Data from a German Observational Study - (NIS HerSCin) [J]. Anticancer research, 2021, 41(1): 485-496. | The research design is inconsistent |  |
| [180] | Seweryn M, Banas T, Augustynska J, Leszczynska A, Potocki P M. - Non-drug related costs of treatment with pertuzumab and trastuzumab in HER2-positive breast cancer patients in Poland [J]. 2024, - 20(- 3): - 189. | The outcome indicators do not conform. |  |
| [181] | Shao Z, Huang T, Fan Z, Wang Y, Yan X, Yang H, et al. 1MO The fixed-dose combination of pertuzumab and trastuzumab for subcutaneous injection (PH FDC SC) in Chinese patients (pts) with HER2-positive early breast cancer (EBC): Primary analysis of the phase III, randomised FDChina study [J]. Annals of Oncology, 2022, 33: S1431. | The research design is inconsistent |  |
| [182] | Simoens S, Vulto A G, Dylst P. - Simulating Costs of Intravenous Biosimilar Trastuzumab vs. Subcutaneous Reference Trastuzumab in Adjuvant HER2-Positive Breast Cancer: A Belgian Case Study [J]. 2021, - 14(- 5). | The type of the research does not match. |  |
| [183] | Stebbing J, Baranau Y, Baryash V, Manikhas A, Moiseyenko V, Dzagnidze G, et al. CT-P6 compared with reference trastuzumab for HER2-positive breast cancer: a randomised, double-blind, active-controlled, phase 3 equivalence trial [J]. The Lancet Oncology, 2017, 18(7): 917‐928. | The research design is inconsistent |  |
| [184] | Stefanou D, Kokkali S, Tripodaki E-S, Drizou M, Magou E, Zylis D, et al. - Subcutaneous Trastuzumab Combined with Pertuzumab and Docetaxel as First-line Treatment of Advanced HER2-positive Breast Cancer [J]. 2018, - 38(- 11): - 6569. | The research design is inconsistent |  |
| [185] | Sun B, Wang Y, Wang H, Zhang X, Li M. - Subcutaneous trastuzumab versus intravenous trastuzumab for treatment of patients with HER2-positive breast cancer: A time, motion and cost-benefit assessment in a day care oncology unit in China [J]. 2023, - 34: - S1477. | The research design is inconsistent |  |
| [186] | Swain S, Barrios C, Basho R, Curigliano G, Harbeck N, Huang C S, et al. INAVO122: a Phase III study of maintenance inavolisib or placebo + pertuzumab + trastuzumab following induction with pertuzumab + trastuzumab + a taxane in patients with PIK3CA-mutated, HER2-positive advanced breast cancer [J]. Cancer Research, 2024, 84(9). | Irrelevant study |  |
| [187] | Swain S M, Baselga J, Kim S B, Ro J, Semiglazov V, Campone M, et al. Pertuzumab, trastuzumab, and docetaxel in HER2-positive metastatic breast cancer [J]. New England journal of medicine, 2015, 372(8): 724‐734. | The research design is inconsistent |  |
| [188] | Swain S M, Tan A, Gianni L, Kümmel S, Dang C, Schneeweiss A, et al. Anaphylaxis and hypersensitivity in trials of intravenous pertuzumab + trastuzumab (PH IV) or the fixed-dose combination of pertuzumab and trastuzumab for subcutaneous injection (PH FDC SC) for HER2-positive breast cancer (BC) [J]. Annals of Oncology, 2021, 32: S419-S420. | Irrelevant study |  |
| [189] | Syrios J, Pappa E, Volakakis N, Grivas A, Alafis J, Manioudaki S, et al. - Real-World Data on Health-Related Quality of Life Assessment in Patients With Breast Cancer Receiving Subcutaneous Trastuzumab [J]. 2018, - 12: - 1178223418758031. | There are no control measures. |  |
| [190] | Tan A R, Im S-A, Mattar A, Colomer R, Stroyakovskii D, Nowecki Z, et al. - Subcutaneous administration of the fixed-dose combination of trastuzumab and pertuzumab in combination with chemotherapy in HER2-positive early breast cancer: Primary analysis of the phase III, multicenter, randomized, open-label, two-arm FeDeriCa study [J]. 2020, - 80(- 4). | The research design is inconsistent |  |
| [191] | Tang Y, Scollard D, Chen P, Wang J, Holloway C, Reilly R M. Imaging of HER2/neu expression in BT-474 human breast cancer xenografts in athymic mice using [(99m)Tc]-HYNIC-trastuzumab (Herceptin) Fab fragments [J]. Nuclear medicine communications, 2005, 26(5): 427-432. | Irrelevant study |  |
| [192] | Thuy N, Duyen T, Nguyen C. - Cost benefits of trastuzumab subcutaneous vs intravenous in treatment of her2-positive breast cancer: A systematic review [J]. 2020, - 29: - 643. | The outcome indicators do not conform |  |
| [193] | Tjalma W, Huizing M T, Papadimitriou K. - The smooth and bumpy road of trastuzumab administration: from intravenous (IV) in a hospital to subcutaneous (SC) at home [J]. 2017, - 9(- 1): - 55. | The research design is inconsistent |  |
| [194] | Tjalma W A A, Van den Mooter T, Mertens T, Bastiaens V, Altintas S, Huizing M T, et al. - Trastuzumab IV <i>versus</i> SC: A time, motion and cost assessment in a lean operating day care oncology unit [J]. 2017, - 77. | The research design is inconsistent |  |
| [195] | Tjalma W A A, Van den Mooter T, Mertens T, Bastiaens V, Huizing M T, Papadimitriou K. Subcutaneous trastuzumab (Herceptin) versus intravenous trastuzumab for the treatment of patients with HER2-positive breast cancer: A time, motion and cost assessment study in a lean operating day care oncology unit [J]. European journal of obstetrics, gynecology, and reproductive biology, 2018, 221: 46-51. | The research design is inconsistent |  |
| [196] | Todorovic V, Durutovic I, Ivanovska A, Zajmovic A. - Subcutaneous Vs Intravenous Administration of Trastuzumab in Her2+Breast Cancer Patients: A Montenegrin Cost-Minimization Analysis [J]. 2017, - 20(- 9): - A443. | The outcome indicators do not conform. |  |
| [197] | Tong Z. Clinical research progress on treatments for HER-2-positive breast cancer [J]. Chinese Journal of Clinical Oncology, 2017, 44(13): 630-634. | The type of the research does not match. |  |
| [198] | Triantafyllidi E, Triantafillidis J K. - Systematic Review on the Use of Biosimilars of Trastuzumab in HER2+ Breast Cancer [J]. 2022, - 10(- 8). | The type of the research does not match. |  |
| [199] | Van den Nest M, Glechner A, Gold M, Gartlehner G. The comparative efficacy and risk of harms of the intravenous and subcutaneous formulations of trastuzumab in patients with HER2-positive breast cancer: a rapid review [J]. Syst Rev, 2019, 8(1): 321. | The type of the research does not match. |  |
| [200] | Vogel C L, Cobleigh M A, Tripathy D, Gutheil J C, Harris L N, Fehrenbacher L, et al. First-line Herceptin monotherapy in metastatic breast cancer [J]. Oncology, 2001, 61 Suppl 2: 37‐42. | Irrelevant study |  |
| [201] | von Minckwitz G, Colleoni M, Kolberg H C, Morales S, Santi P, Tomasevic Z, et al. Efficacy and safety of ABP 980 compared with reference trastuzumab in women with HER2-positive early breast cancer (LILAC study): a randomised, double-blind, phase 3 trial [J]. The Lancet Oncology, 2018, 19(7): 987‐998. | Irrelevant study |  |
| [202] | Waks A, Graham N, Chen E, Frey A M, Attaya V, Abbass I, et al. - Patient (pt) time burden with IV vs subcutaneous (SC) administration of trastuzumab/pertuzumab (HP): A time and motion (T plus M) substudy of a single arm phase II trial of adjuvant endocrine therapy plus HP for stage I HER2+breast cancer [J]. 2024, - 84(- 9). | The research design is inconsistent. |  |
| [203] | Waks A G, Chen E L, Graham N, Frey A M, Almeida K, Attaya V, et al. - Subcutaneous vs Intravenous Trastuzumab/Pertuzumab: A Time and Motion Substudy of a Phase II Trial of Adjuvant Trastuzumab/Pertuzumab for Stage I HER2+ Breast Cancer (ADEPT trial) [J]. 2024: - OP2400021. | The research design is inconsistent. |  |
| [204] | Waller C F, Möbius J, Fuentes-Alburo A. Intravenous and subcutaneous formulations of trastuzumab, and trastuzumab biosimilars: implications for clinical practice [J]. British journal of cancer, 2021, 124(8): 1346-1352. | The research type does not match. |  |
| [205] | Waller C F, Vutikullird A, Lawrence T E, Shaw A, Liu M S, Baczkowski M, et al. A pharmacokinetics phase 1 bioequivalence study of the trastuzumab biosimilar MYL-1401O vs. EU-trastuzumab and US-trastuzumab [J]. British journal of clinical pharmacology, 2018, 84(10): 2336‐2343. | Irrelevant study |  |
| [206] | Wang B, Deng R, Hennig S, Badovinac Crnjevic T, Kaewphluk M, Kågedal M, et al. Population pharmacokinetic and exploratory exposure-response analysis of the fixed-dose combination of pertuzumab and trastuzumab for subcutaneous injection in patients with HER2-positive early breast cancer in the FeDeriCa study [J]. Cancer chemotherapy and pharmacology, 2021, 88(3): 499-512. | Irrelevant study |  |
| [207] | Wang Z, Li X, Yu X, Jin F. Important research progress in clinical practice for early breast cancer in 2023 [J]. China Oncology, 2024, 34(2): 151-160. | Irrelevant study |  |
| [208] | Wong C Y, Leung R, Kwok G W, Tsang J, Li B, Yau T, et al. Safety and tolerability of subcutaneous trastuzumab and intravenous pertuzumab as adjuvant treatment for HER2 positive breast cancer: a pilot study [J]. Postgraduate medical journal, 2022, 98(1163): 666-669. | The research design is inconsistent |  |
| [209] | Woodward N, De Boer R H, Redfern A, Von Neumann-Cosel V, Heath R M, Beith J. - An open-label, multicentre, phase IIIb study with intravenous administration of pertuzumab, subcutaneous trastuzumab, and a taxane in patients with HER2-positive metastatic breast cancer (SAPPHIRE) [J]. 2015, - 75. | The research design is inconsistent |  |
| [210] | Woodward N, De Boer R H, Redfern A, White M, Young J, Truman M, et al. Results From the First Multicenter, Open-label, Phase IIIb Study Investigating the Combination of Pertuzumab With Subcutaneous Trastuzumab and a Taxane in Patients With HER2-positive Metastatic Breast Cancer (SAPPHIRE) [J]. Clinical breast cancer, 2019, 19(3): 216-224. | The research design is inconsistent |  |
| [211] | Wyrwicz L, Sanchez C A R, Sanchez-Rovira P, Lewis S, Sandschafer D, San T. - Real-world clinical scenarios during introduction of trastuzumab biosimilar for HER2-positive breast cancer in the European Union [J]. 2024, - 20(- 13): - 832. | The research design is inconsistent |  |
| [212] | Yu A F, Ferraro E, Liu J E, Advani P, Herrmann J, Yang A, et al. - Home Cardiac Surveillance with Artificial Intelligence Digital Patient Monitoring during Treatment with Pertuzumab, Trastuzumab and Hyaluronidase-Zzxf for Her2-Positive Breast Cancer (Harriet): Study Design and Rationale [J]. 2022, - 79(- 9): - 2004. | The research design is inconsistent. |  |
| [213] | Zambetti M, Montemurro F, Morandi P, Zamagni C, Brandes A A, Bisagni G, et al. Safety profile of subcutaneous trastuzumab for the treatment of patients with HER2-positive early or locally advanced breast cancer: primary analysis of the SCHEARLY study [J]. European journal of cancer (Oxford, England : 1990), 2018, 105: 61-70. | No control measures |  |
| [214] | Zhou Z, Vaidyanathan G, McDougald D, Kang C M, Balyasnikova I, Devoogdt N, et al. Fluorine-18 Labeling of the HER2-Targeting Single-Domain Antibody 2Rs15d Using a Residualizing Label and Preclinical Evaluation [J]. Molecular imaging and biology, 2017, 19(6): 867-877. | Irrelevant study |  |
| [215] | Ziegengeist J L, Tan A R. - A Clinical Review of Subcutaneous Trastuzumab and the Fixed-Dose Combination of Pertuzumab and Trastuzumab for Subcutaneous Injection in the Treatment of HER2-Positive Breast Cancer [J]. 2024. | The type of the research does not match. |  |
| [216] | Elsamany S A, Jafal M, Hassanin F. Pharmaco-economic assessment of subcutaneous compared to intravenous trastuzumab in HER2-positive breast cancer patients: A single institution experience [J]. 2020, - 38(- 15). | The outcome indicators do not conform. |  |
| [217] | Gligorov J, Ataseven B, Verrill M, De Laurentiis M, Jung K H, Azim H A, et al. Safety and tolerability of subcutaneous trastuzumab for the adjuvant treatment of human epidermal growth factor receptor 2-positive early breast cancer: SafeHer phase III study's primary analysis of 2573 patients [J]. European journal of cancer (Oxford, England : 1990), 2017, 82: 237-246. | No control measures |  |
| [218] | Gligorov J, Curigliano G, Mueller V, Knoop A, Jenkins V, Verma S, et al. - Switching between intravenous and subcutaneous trastuzumab: Safety results from the PrefHer trial [J]. 2017, - 34: - 95. | The research design is not in line with. |  |
| [219] | González García J, Gutiérrez Nicolás F, Ramos Díaz R, Nazco Casariego G J, Viña Romero M M, Llabres Martinez M, et al. Pharmacokinetics of Trastuzumab After Subcutaneous and Intravenous Administration in Obese Patients [J]. The Annals of pharmacotherapy, 2020, 54(8): 775-779. | The research population does not match. |  |
| [220] | Valachis A, Sundqvist M, Carlsson L, Li B, Chiesa F, Uhde M, et al. Use of subcutaneous and intravenous trastuzumab: real-world experience from three hospitals in Sweden [J]. Future oncology (London, England), 2019, 15(23): 2733-2741. | The research design is not in line with. |  |
| [221] | Jackisch C, Müller V, Dall P, Neumeister R, Park-Simon T W, Ruf-Dördelmann A, et al. Subcutaneous trastuzumab for HER2-positive breast cancer - Evidence and practical experience in 7 German centers [J]. Geburtshilfe und Frauenheilkunde, 2015, 75(6): 566-573. | No control measures |  |
| [222] | López M A A, Samanes M A S, Tena I P, Alonso E F, Turlan V C, Sanchez M J C, et al. Switching from intravenous to subcutaneous formulation of trastuzumab: Costs and safety [J]. European Journal of Hospital Pharmacy, 2017, 24: A57. | The research design is not in line with. |  |
| [223] | Lopez-Vivanco G, Salvador J, Diez R, López D, De Salas-Cansado M, Navarro B, et al. Cost minimization analysis of treatment with intravenous or subcutaneous trastuzumab in patients with HER2-positive breast cancer in Spain [J]. Clinical & translational oncology : official publication of the Federation of Spanish Oncology Societies and of the National Cancer Institute of Mexico, 2017, 19(12): 1454-1461. | The outcome indicators do not conform. |  |
| [224] | Möbus V, Mahlberg R, Janni W, Tomé O, Marmé F, Forstbauer H, et al. Pharmacokinetic results of a subcutaneous injection of trastuzumab into the thigh versus into the abdominal wall in patients with HER2-positive primary breast cancer (BC) treated within the neo-/adjuvant GAIN-2 study [J]. Cancer Research, 2018, 78(4). | The contrast measures do not conform. |  |
| [225 | Olsen J, Jensen K F, Olesen D S, Knoop A. Costs of subcutaneous and intravenous administration of trastuzumab for patients with HER2-positive breast cancer [J]. Journal of comparative effectiveness research, 2018, 7(5): 411-419. | The outcome indicators do not conform. |  |
| [226] | Swain S M, Tan A R, Gianni L, Kuemmel S, Dang C T, Schneeweiss A, et al. Incidence and severity of anaphylaxis and hypersensitivity in trials of intravenous pertuzumab plus trastuzumab or the fixed-dose combination of pertuzumab and trastuzumab for subcutaneous injection for HER2-positive breast cancer [J]. European journal of cancer (Oxford, England : 1990), 2023, 178: 70-81. | The outcome indicators do not conform. |  |
| [227] | Wynne C, Harvey V, Schwabe C, Waaka D, McIntyre C, Bittner B. Comparison of subcutaneous and intravenous administration of trastuzumab: a phase I/Ib trial in healthy male volunteers and patients with HER2-positive breast cancer [J]. Journal of clinical pharmacology, 2013, 53(2): 192-201. | The research population does not match. |  |
| [228] | Petrakova K, Melichar B, Bortlicek Z, Hejduk K. Preference of trastuzumab administration route (intravenous or subcutaneous) in patients in the Czech Republic. Cross-sectional study on 429 patients [J]. Cancer Research, 2017, 77(4). | The type of the research does not match. |  |
| [229] | Pivot X, Gligorov J, Müller V, Curigliano G, Knoop A, Verma S, et al. Patients' preferences for subcutaneous trastuzumab versus conventional intravenous infusion for the adjuvant treatment of HER2-positive early breast cancer: final analysis of 488 patients in the international, randomized, two-cohort PrefHer study [J]. Annals of oncology : official journal of the European Society for Medical Oncology, 2014, 25(10): 1979-1987. | Be included in the study |  |
| [230] | Pivot X, Spano J P, Espie M, Cottu P, Jouannaud C, Pottier V, et al. - Patients' preference of trastuzumab administration (subcutaneous versus intravenous) in HER2-positive metastatic breast cancer: Results of the randomised MetaspHer study [J]. 2017, - 82: - 236. | Be included in the study |  |
| [231] | Pivot X, Verma S, Fallowfield L, Müller V, Lichinitser M, Jenkins V, et al. Efficacy and safety of subcutaneous trastuzumab and intravenous trastuzumab as part of adjuvant therapy for HER2-positive early breast cancer: Final analysis of the randomised, two-cohort PrefHer study [J]. European journal of cancer (Oxford, England : 1990), 2017, 86: 82-90. | Be included in the study |  |
| [232] | Tan A R, Im S A, Mattar A, Colomer R, Stroyakovskii D, Nowecki Z, et al. Fixed-dose combination of pertuzumab and trastuzumab for subcutaneous injection plus chemotherapy in HER2-positive early breast cancer (FeDeriCa): a randomised, open-label, multicentre, non-inferiority, phase 3 study [J]. The Lancet Oncology, 2021, 22(1): 85-97. | Be included in the study |  |
| [233] | Ismael G, Hegg R, Muehlbauer S, Heinzmann D, Lum B, Kim S B, et al. Subcutaneous versus intravenous administration of (neo)adjuvant trastuzumab in patients with HER2-positive, clinical stage I-III breast cancer (HannaH study): a phase 3, open-label, multicentre, randomised trial [J]. The Lancet Oncology, 2012, 13(9): 869‐878. | Be included in the study |  |
| [234] | Jackisch C, Stroyakovskiy D, Pivot X, Ahn J S, Melichar B, Chen S C, et al. Subcutaneous vs Intravenous Trastuzumab for Patients With ERBB2-Positive Early Breast Cancer: Final Analysis of the HannaH Phase 3 Randomized Clinical Trial [J]. JAMA oncology, 2019, 5(5): e190339. | Be included in the study |  |
| [235] | O'Shaughnessy J, Sousa S, Cruz J, Fallowfield L, Auvinen P, Pulido C, et al. Preference for the fixed-dose combination of pertuzumab and trastuzumab for subcutaneous injection in patients with HER2-positive early breast cancer (PHranceSCa): A randomised, open-label phase II study [J]. European Journal of Cancer, 2021, 152: 223-232. | Be included in the study |  |
| [236] | Pellegrino B, Tommasi C, Serra O, Gori S, Cretella E, Ambroggi M, et al. Randomized, open-label, phase II, biomarker study of immune-mediated mechanism of action of neoadjuvant subcutaneous trastuzumab in patients with locally advanced, inflammatory, or early HER2-positive breast cancer-Immun-HER trial (GOIRC-01-2016) [J]. Journal for immunotherapy of cancer, 2023, 11(11). | Be included in the study |  |
| [237] | Pivot X, Gligorov J, Müller V, Barrett-Lee P, Verma S, Knoop A, et al. Preference for subcutaneous or intravenous administration of trastuzumab in patients with HER2-positive early breast cancer (PrefHer): an open-label randomised study [J]. The Lancet Oncology, 2013, 14(10): 962-970. | Be included in the study |  |

# Table S3. GRADE Quality Assessment Table

| Outcome | No. of studies | Evaluation of evidence quality | | | | | sample size | | Evidence level |
| --- | --- | --- | --- | --- | --- | --- | --- | --- | --- |
|  |  | risk of bias | inconsistency | indirectness | imprecision | publication bias | T | C |  |
| pCR | 2 | 0 | 0 | 0 | -1^a^ | 0 | 508 | 515 | moderate |
| EFS | 2 | 0 | 0 | 0 | -1^a^ | 0 | 538 | 536 | moderate |
| AE | 8 | 0 | 0 | 0 | 0 | 0 | 2094 | 2095 | high |
| SAE | 4 | 0 | 0 | 0 | -1^a^ | 0 | 1044 | 1047 | moderate |
| Preference | 4 | 0 | -2^b^ | 0 | 0 | 0 | 955 | 955 | Low |

Note: ^a^ The confidence interval is wide and crosses the invalid line;^b^ The I^2^ statistic exceeds 50%, indicating the presence of significant heterogeneity.

# Table S4.Total drug costs of different trastuzumab formulations over 18 treatment cycles(CNY)

|  | SC Trastuzumab 600 mg/vial | IV Originator (Herceptin® 440 mg/vial) | IV Biosimilar (Hanquyou® 150 mg/vial) | IV Biosimilar (Saitu® 150 mg/vial) | IV Biosimilar (Anqutuo® 150 mg/vial) |
| --- | --- | --- | --- | --- | --- |
| Unit price(CNY) | 4800 | 5500 | 1688 | 1587 | 1180 |
| Initial dose cost(Cycle 1) | 4800 | 11000 | 6752 | 6348 | 4720 |
| Subsequent doses  (Cycles 2-18) | 81600 | 82500 | 86088 | 80937 | 60180 |
| Total cost(18 cycles) | 86400 | 93500 | 92840 | 87285 | 64900 |

# Table S5. Estimation of the Consumable Costs for Different Dosage Forms of Trastuzumab(CNY)

|  | Subcutaneous administration of trastuzumab | | Intravenous administration of trastuzumab | |
| --- | --- | --- | --- | --- |
|  | quantity | Price | quantity | Price |
| Sterile Water for Injection, 2 mL | 0 | 0 | 12 | 1.44 |
| 0.9% Sodium Chloride Injection, 250 mL: Contains 2.25 g of sodium chloride | 0 | 0 | 1 | 4.30 |
| Disposable sterile infusion set with needle | 0 | 0 | 1 | 0.43 |
| Prefilled catheter irrigation device | 0 | 0 | 1 | 1.47 |
| Disposable sterile dressing (6cm * 7cm) | 0 | 0 | 1 | 0.53 |
| Closed-type intravenous indwelling needle with anti-needlestick injury protection, 24G, needle-free three-way connector with end cap | 0 | 0 | 1 | 5.47 |
| Preparation of Antitumor Agents | 1 | 18.8 | 1 | 18.8 |
| Total cost of single-use consumables |  | 18.8 |  | 32.44 |
| Total cost(18 cycles) |  | 338.4 |  | 538.92 |

# Table S6. Administration and Service Costs for Subcutaneous and Intravenous Trastuzumab

| Cost Component | SC (CNY) | IV (CNY) |
| --- | --- | --- |
| **Cost per administration cycle** |  |  |
| Consultation fee | 15.5 | 15.5 |
| Nursing fee | 20 | 20 |
| Intravenous infusion fee | 0 | 8 |
| Subcutaneous injection fee | 3 | 0 |
| Total per cycle | 38.5 | 43.5 |
| **Cumulative cost over treatment course** |  |  |
| First administration (Cycle 1) | 38.5 | 43.5 |
| Subsequent administrations (Cycles 2-18) | 654.5 | 739.5 |
| Total for 18 cycles | 693 | 783 |

# Table S7. Monitoring and Laboratory Costs for Trastuzumab Treatment

| Test Category | Specific Test | Unit Cost (CNY) |
| --- | --- | --- |
| Hematology and biochemistry |  |  |
|  | Complete blood count | 25.0 |
|  | Hepatic function panel | 59.7 |
|  | Renal function panel | 25.9 |
|  | Serum tumor markers | 202.0 |
|  | Cardiac biomarkers | 32.9 |
|  | Hormonal panel (6 parameters) | 180.0 |
| Cardiac assessment |  |  |
|  | 12-lead electrocardiogram | 25.0 |
|  | Cardiac function test | 46.0 |
|  | Transthoracic echocardiography | 105.0 |
| Imaging studies |  |  |
|  | Bilateral mammography | 194.5 |
|  | Breast MRI (non-contrast) | 594.0 |
|  | Breast and axillary ultrasound | 66.5 |
|  | Abdominal ultrasound | 74.1 |
|  | Chest CT (contrast-enhanced) | 633.0 |
|  | Abdominal CT (contrast-enhanced) | 1,590.0 |
| Cumulative monitoring costs |  |  |
|  | Baseline assessment (Cycle 1) | 3,853.6 |
|  | Follow-up assessments (Cycles 2-18) | 9,312.0 |
|  | Total (18 cycles) | 13,165.6 |
| Note: Monitoring costs are identical for both SC and IV trastuzumab as they follow standardized clinical protocols. Baseline assessment (Cycle 1) includes comprehensive testing. Follow-up assessments (Cycles 2-18) are performed at reduced frequency according to clinical guidelines. All costs are based on the hospital fee schedule and represent actual charges in 2025. | | |

# Figure 1. Risk of Bias Assessment Diagram for Randomized Controlled Trials


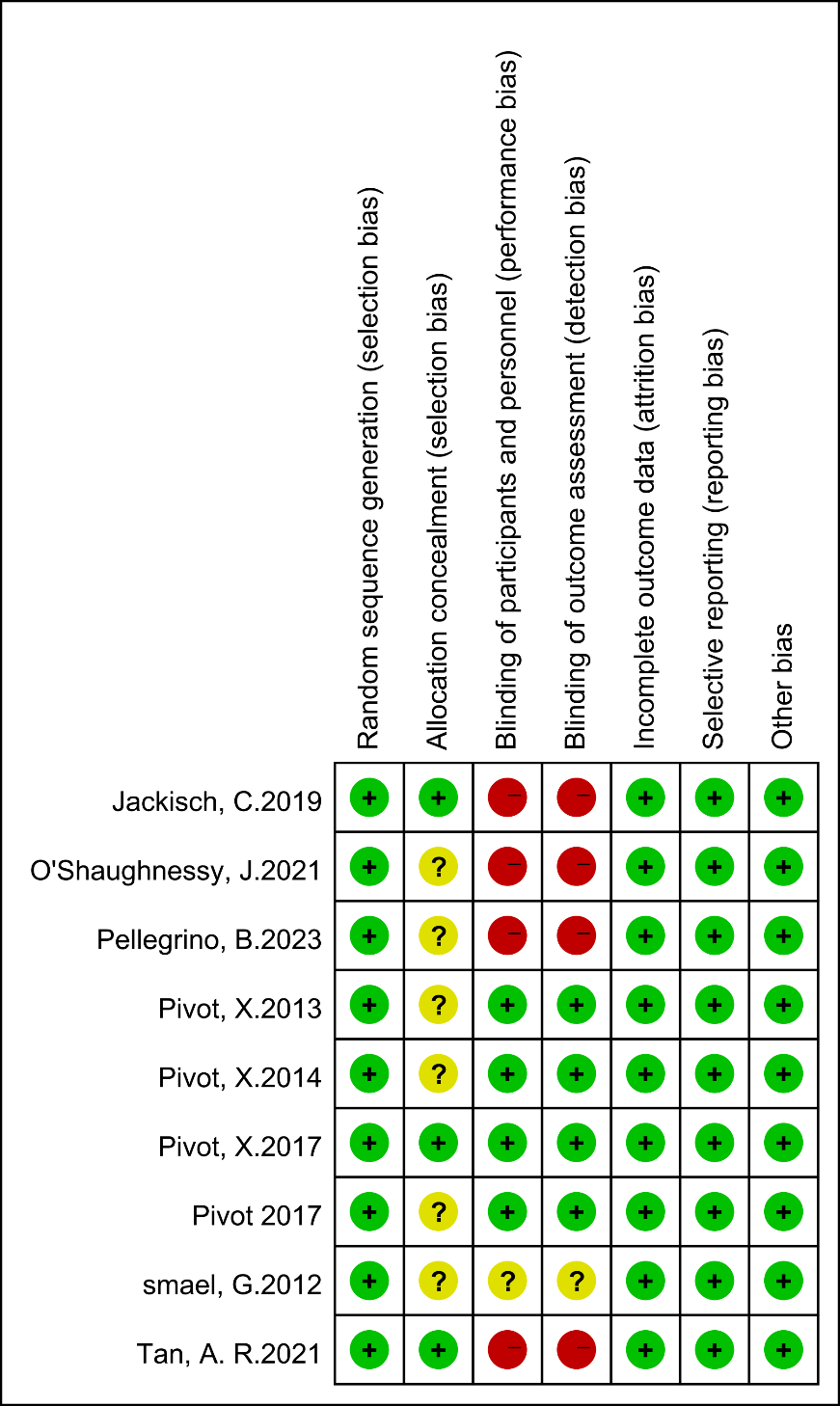


# Figure 2. One-Way Sensitivity Analysis: Subcutaneous Trastuzumab


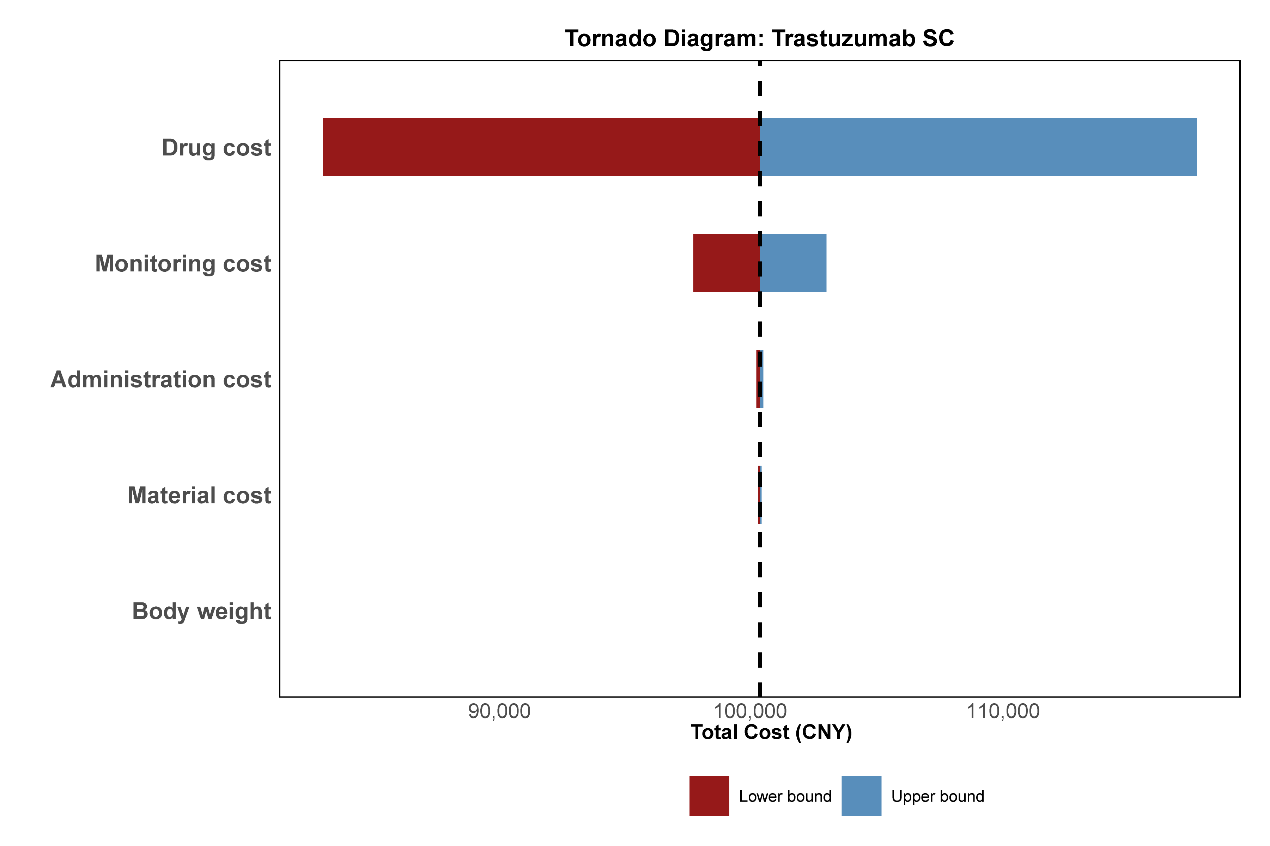


# Figure 3. One-Way Sensitivity Analysis: Intravenous Originator Trastuzumab (Herceptin®)


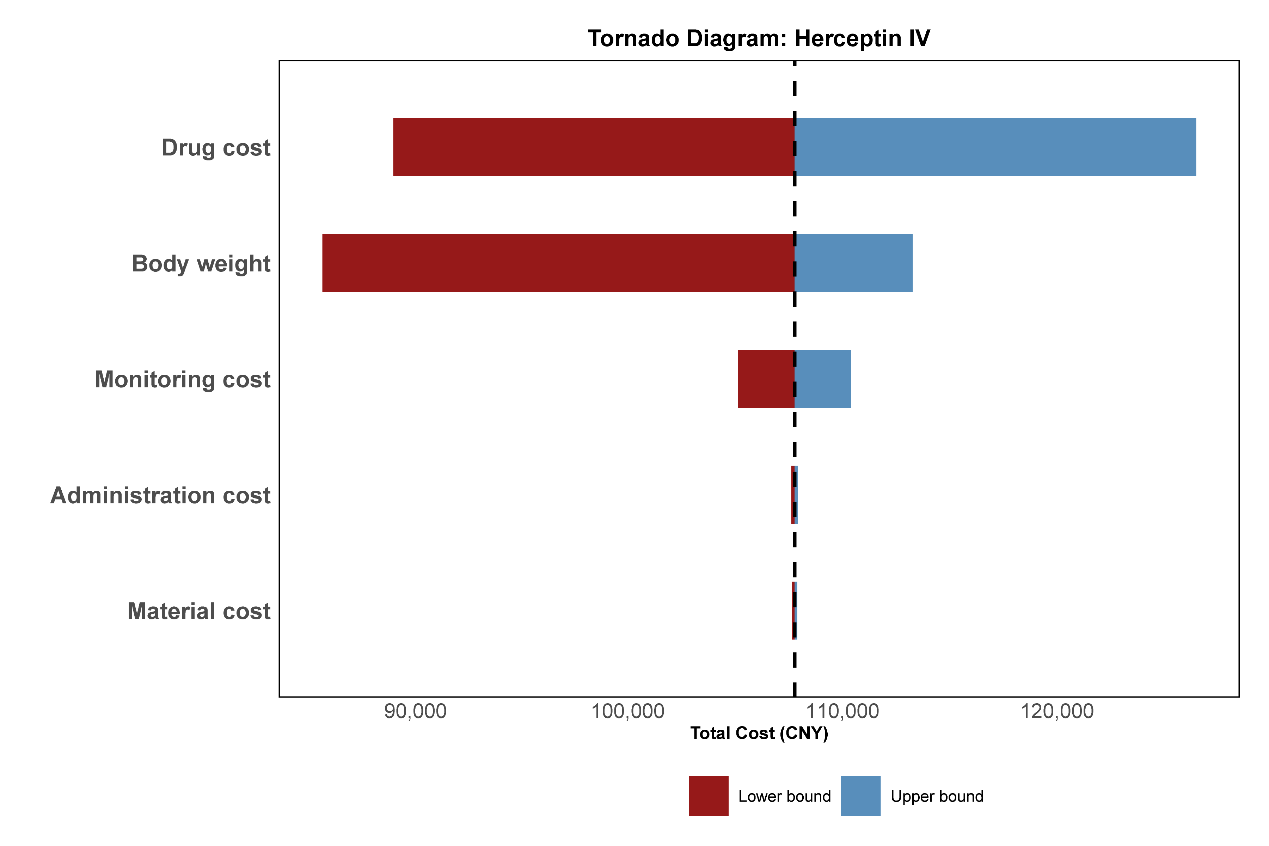


# Figure 4. One-Way Sensitivity Analysis: Intravenous Originator Trastuzumab (Hanquyou®)


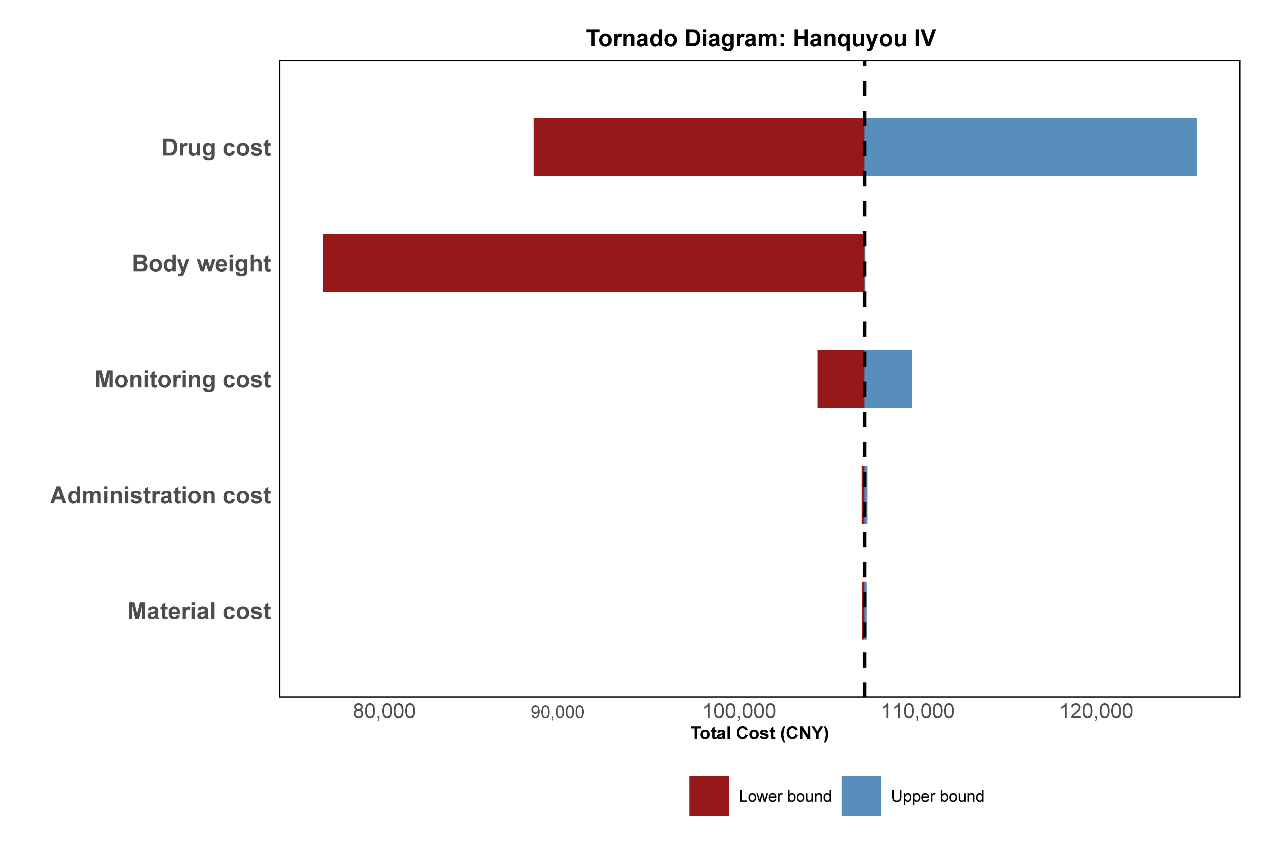


# Figure 5. One-Way Sensitivity Analysis: Intravenous Originator Trastuzumab(Saitu®)


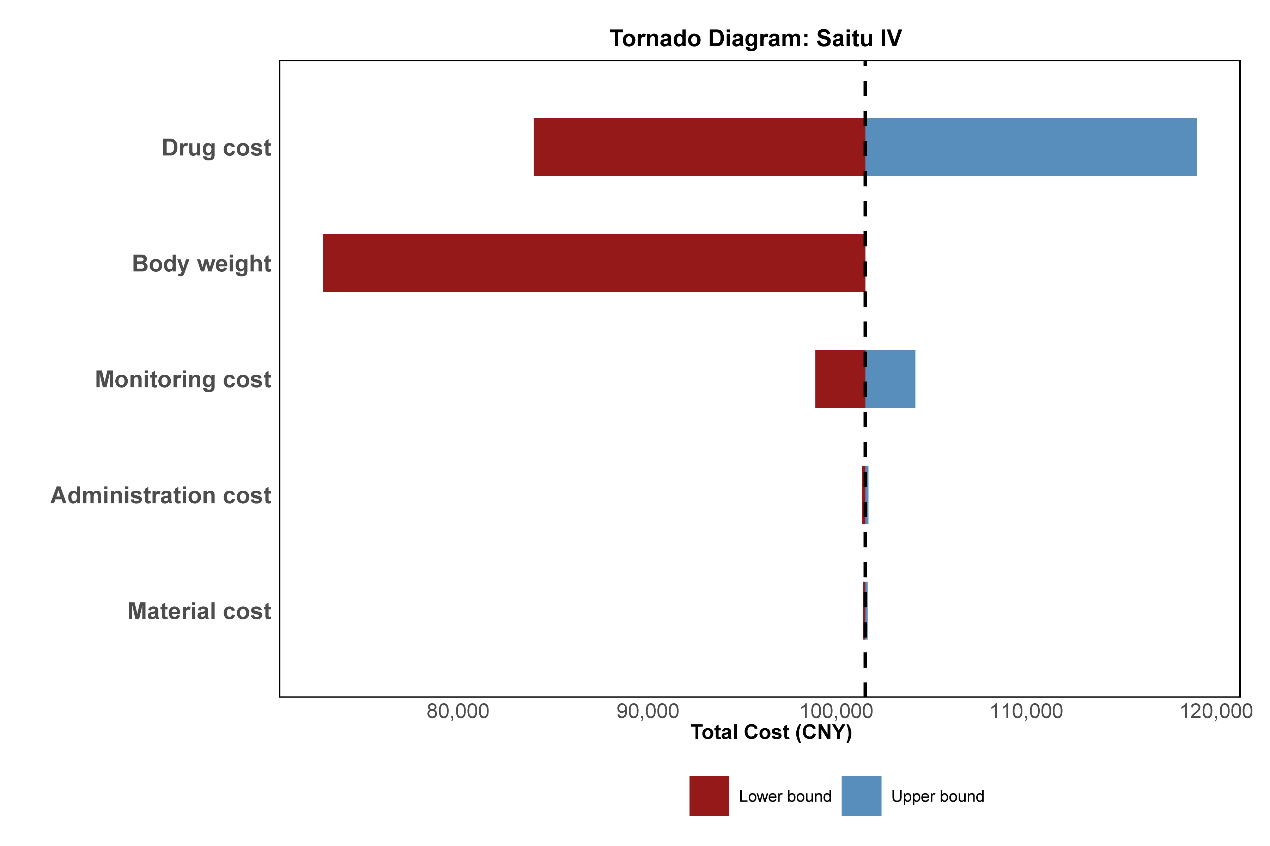


# Figure 6. One-Way Sensitivity Analysis: Intravenous Originator Trastuzumab (Anqutuo®)


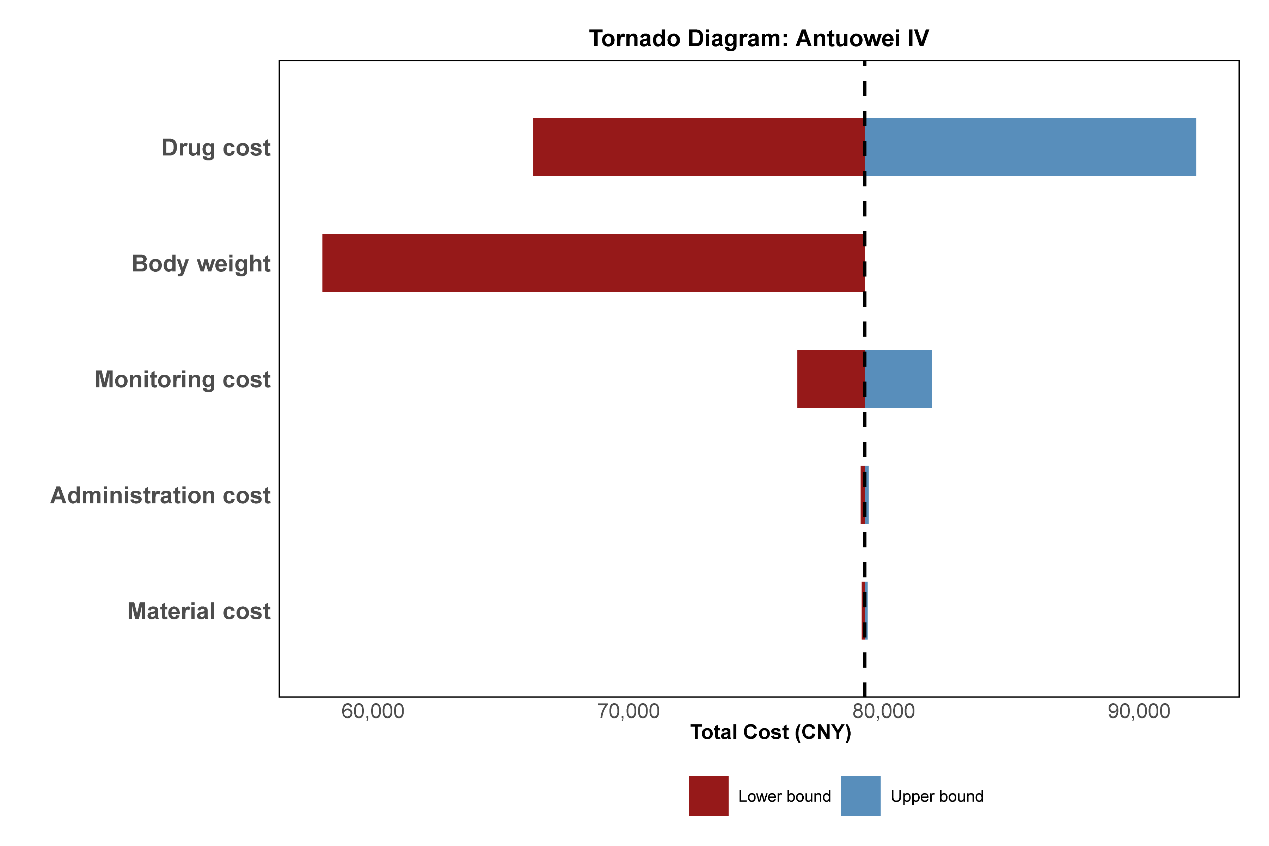


# PRISMA checklist

| **Section and Topic** | **Item #** | **Checklist item** | **Location where item is reported** |
| --- | --- | --- | --- |
| **TITLE** | | |  |
| Title | 1 | Identify the report as a systematic review. | Title page |
| **ABSTRACT** | | |  |
| Abstract | 2 | See the PRISMA 2020 for Abstracts checklist. | Abstract |
| **INTRODUCTION** | | |  |
| Rationale | 3 | Describe the rationale for the review in the context of existing knowledge. | Introduction |
| Objectives | 4 | Provide an explicit statement of the objective(s) or question(s) the review addresses. | Introduction |
| **METHODS** | | |  |
| Eligibility criteria | 5 | Specify the inclusion and exclusion criteria for the review and how studies were grouped for the syntheses. | Methods  (Section 2.1) |
| Information sources | 6 | Specify all databases, registers, websites, organisations, reference lists and other sources searched or consulted to identify studies. Specify the date when each source was last searched or consulted. | Methods  (Section 2.2)  Supplementary Material Table S1 |
| Search strategy | 7 | Present the full search strategies for all databases, registers and websites, including any filters and limits used. | Methods  (Section 2.2)  Supplementary Material Table S1 |
| Selection process | 8 | Specify the methods used to decide whether a study met the inclusion criteria of the review, including how many reviewers screened each record and each report retrieved, whether they worked independently, and if applicable, details of automation tools used in the process. | Methods  (Section 2.3) |
| Data collection process | 9 | Specify the methods used to collect data from reports, including how many reviewers collected data from each report, whether they worked independently, any processes for obtaining or confirming data from study investigators, and if applicable, details of automation tools used in the process. | Methods  (Section 2.3) |
| Data items | 10a | List and define all outcomes for which data were sought. Specify whether all results that were compatible with each outcome domain in each study were sought (e.g. for all measures, time points, analyses), and if not, the methods used to decide which results to collect. | Methods  (Section 2.1.1)  Table1 |
|  | 10b | List and define all other variables for which data were sought (e.g. participant and intervention characteristics, funding sources). Describe any assumptions made about any missing or unclear information. | Methods  (Section 2.3)  Table1 |
| Study risk of bias assessment | 11 | Specify the methods used to assess risk of bias in the included studies, including details of the tool(s) used, how many reviewers assessed each study and whether they worked independently, and if applicable, details of automation tools used in the process. | Methods  (Section 2.4) |
| Effect measures | 12 | Specify for each outcome the effect measure(s) (e.g. risk ratio, mean difference) used in the synthesis or presentation of results. | Methods  (Section 2.5) |
| Synthesis methods | 13a | Describe the processes used to decide which studies were eligible for each synthesis (e.g. tabulating the study intervention characteristics and comparing against the planned groups for each synthesis (item #5)). | Results  (Section 3.1) |
|  | 13b | Describe any methods required to prepare the data for presentation or synthesis, such as handling of missing summary statistics, or data conversions. | Methods  (Section 2.5) |
|  | 13c | Describe any methods used to tabulate or visually display results of individual studies and syntheses. | Methods  (Section 2.3)  Section 2.5) |
|  | 13d | Describe any methods used to synthesize results and provide a rationale for the choice(s). If meta-analysis was performed, describe the model(s), method(s) to identify the presence and extent of statistical heterogeneity, and software package(s) used. | Methods  (Section 2.5) |
|  | 13e | Describe any methods used to explore possible causes of heterogeneity among study results (e.g. subgroup analysis, meta-regression). | Methods  (Section 2.5) |
|  | 13f | Describe any sensitivity analyses conducted to assess robustness of the synthesized results. | Methods  (Section 2.5)  (Section 2.7.3) |
| Reporting bias assessment | 14 | Describe any methods used to assess risk of bias due to missing results in a synthesis (arising from reporting biases). | Methods  (Section 2.5) |
| Certainty assessment | 15 | Describe any methods used to assess certainty (or confidence) in the body of evidence for an outcome. | Methods  (Section 2.6)  Supplementary Material  Table S3 |
| **RESULTS** | | |  |
| Study selection | 16a | Describe the results of the search and selection process, from the number of records identified in the search to the number of studies included in the review, ideally using a flow diagram. | Results  (Section 3.1)  Figure1  Supplementary Material Table S1  Table S2 |
|  | 16b | Cite studies that might appear to meet the inclusion criteria, but which were excluded, and explain why they were excluded. | Figure1  Supplementary Material Table S3 |
| Study characteristics | 17 | Cite each included study and present its characteristics. | Table1 |
| Risk of bias in studies | 18 | Present assessments of risk of bias for each included study. | Results  (Section 3.3)  Supplementary Material Figure 1 |
| Results of individual studies | 19 | For all outcomes, present, for each study: (a) summary statistics for each group (where appropriate) and (b) an effect estimate and its precision (e.g. confidence/credible interval), ideally using structured tables or plots. | Results  (Section 3.4)  Figure2- Figure6 |
| Results of syntheses | 20a | For each synthesis, briefly summarise the characteristics and risk of bias among contributing studies. | Results  (Section 3.3)  Supplementary Material Figure 1 |
|  | 20b | Present results of all statistical syntheses conducted. If meta-analysis was done, present for each the summary estimate and its precision (e.g. confidence/credible interval) and measures of statistical heterogeneity. If comparing groups, describe the direction of the effect. | Results  (Section 3.4) |
|  | 20c | Present results of all investigations of possible causes of heterogeneity among study results. | Results  (Section 3.4.5)  Discussion |
|  | 20d | Present results of all sensitivity analyses conducted to assess the robustness of the synthesized results. | Results  (Section 3.4.5)  (Section 3.7.3) |
| Reporting biases | 21 | Present assessments of risk of bias due to missing results (arising from reporting biases) for each synthesis assessed. | - |
| Certainty of evidence | 22 | Present assessments of certainty (or confidence) in the body of evidence for each outcome assessed. | Results  (Section 3.6)  Table S3 |
| **DISCUSSION** | | |  |
| Discussion | 23a | Provide a general interpretation of the results in the context of other evidence. | Discussion |
|  | 23b | Discuss any limitations of the evidence included in the review. | Discussion |
|  | 23c | Discuss any limitations of the review processes used. | Discussion |
|  | 23d | Discuss implications of the results for practice, policy, and future research. | Discussion |
| **OTHER INFORMATION** | | |  |
| Registration and protocol | 24a | Provide registration information for the review, including register name and registration number, or state that the review was not registered. | Abstract  Methods  (Section 2.2) |
|  | 24b | Indicate where the review protocol can be accessed, or state that a protocol was not prepared. | - |
|  | 24c | Describe and explain any amendments to information provided at registration or in the protocol. | - |
| Support | 25 | Describe sources of financial or non-financial support for the review, and the role of the funders or sponsors in the review. | Funding |
| Competing interests | 26 | Declare any competing interests of review authors. | Competing interests |
| Availability of data, code and other materials | 27 | Report which of the following are publicly available and where they can be found: template data collection forms; data extracted from included studies; data used for all analyses; analytic code; any other materials used in the review. | Availability of data and materials |
